# VirFinder: a novel *k*-mer based tool for identifying viral sequences from assembled metagenomic data

**DOI:** 10.1186/s40168-017-0283-5

**Published:** 2017-07-06

**Authors:** Jie Ren, Nathan A. Ahlgren, Yang Young Lu, Jed A. Fuhrman, Fengzhu Sun

**Affiliations:** 1 Molecular and Computational Biology Program, University of Southern California, 1050 Childs Way, Los Angeles, CA 90089 USA; 2 Department of Biological Sciences, University of Southern California, 3616 Trousdale Pkwy, Los Angeles, CA 90089 USA; 3 Center for Computational Systems Biology, Fudan University, 200433 Shanghai, China; 4 Present address: Biology Department, Clark University, 950 Main St, Worcester, MA 01610 USA

**Keywords:** Metagenome, Virus, *k*-mer, Human gut, Liver cirrhosis

## Abstract

**Background:**

Identifying viral sequences in mixed metagenomes containing both viral and host contigs is a critical first step in analyzing the viral component of samples. Current tools for distinguishing prokaryotic virus and host contigs primarily use gene-based similarity approaches. Such approaches can significantly limit results especially for short contigs that have few predicted proteins or lack proteins with similarity to previously known viruses.

**Methods:**

We have developed VirFinder, the first *k*-mer frequency based, machine learning method for virus contig identification that entirely avoids gene-based similarity searches. VirFinder instead identifies viral sequences based on our empirical observation that viruses and hosts have discernibly different *k*-mer signatures. VirFinder’s performance in correctly identifying viral sequences was tested by training its machine learning model on sequences from host and viral genomes sequenced before 1 January 2014 and evaluating on sequences obtained after 1 January 2014.

**Results:**

VirFinder had significantly better rates of identifying true viral contigs (true positive rates (TPRs)) than VirSorter, the current state-of-the-art gene-based virus classification tool, when evaluated with either contigs subsampled from complete genomes or assembled from a simulated human gut metagenome. For example, for contigs subsampled from complete genomes, VirFinder had 78-, 2.4-, and 1.8-fold higher TPRs than VirSorter for 1, 3, and 5 kb contigs, respectively, at the same false positive rates as VirSorter (0, 0.003, and 0.006, respectively), thus VirFinder works considerably better for small contigs than VirSorter. VirFinder furthermore identified several recently sequenced virus genomes (after 1 January 2014) that VirSorter did not and that have no nucleotide similarity to previously sequenced viruses, demonstrating VirFinder’s potential advantage in identifying novel viral sequences. Application of VirFinder to a set of human gut metagenomes from healthy and liver cirrhosis patients reveals higher viral diversity in healthy individuals than cirrhosis patients. We also identified contig bins containing crAssphage-like contigs with higher abundance in healthy patients and a putative *Veillonella* genus prophage associated with cirrhosis patients.

**Conclusions:**

This innovative *k*-mer based tool complements gene-based approaches and will significantly improve prokaryotic viral sequence identification, especially for metagenomic-based studies of viral ecology.

**Electronic supplementary material:**

The online version of this article (doi:10.1186/s40168-017-0283-5) contains supplementary material, which is available to authorized users.

## Background

Viruses are the most abundant biological entities with more than 10^31^ particles on Earth, most of which are viruses that infect bacteria and archaea (prokaryotes) [1]. Viruses infect and replicate within host cells, and through these infective interactions, they play important roles in controlling bacterial population size, altering host metabolism, and have broader impacts on the functions of microbial communities, such as human gut, soil, and ocean microbiomes [2]. For example, viruses in the human gut microbiome have been found to profoundly influence inflammatory bowel disease and severe acute malnutrition [3, 4]. In aquatic and soil habitats, viruses also have important roles in affecting the biogeochemical functioning of their hosts [5].

However, our understanding of virus-host interactions for large portions of viral communities has been limited due to the difficulties of using traditional virus isolation techniques, especially for those that infect uncultivable hosts. Isolation approaches have to date only yielded a small portion of known viral diversity–viruses have been isolated on less than 15% of known phyla of prokaryotic hosts (based on data in [6]). While the term virus broadly includes those that infect prokaryotic and eukaryotic hosts, throughout we use the term virus (and provirus in the case of integrated viruses), to refer to viruses that infect bacteria or archaea (the focus of this study) rather than the terms phage or bacteriophage, which specifically refer to viruses that infect bacteria. Metagenomic studies using high throughput sequencing technology can now generate massive amounts of short read sequences from prokaryotic cells in microbial communities regardless of cultivability of the cells, and viruses are inevitably captured at the same time in these samples. Many metagenomic studies specifically focus on selectively capturing and sequencing viral particles, but sequencing cellular fraction samples frequently recover viral sequences along with prokaryotic host sequences. For example, viral sequences were estimated to comprise 4–17% of human gut prokaryote metagenomes [7]. Viral sequences found in cellular prokaryotic samples will include lysogenic viruses integrated into prokaryotic host genomes, or proviruses, as well as viral DNA within actively infected cells and those outside cells but still collected by the sampling method. Likewise, single cell sequencing methods can sometimes simultaneously capture host and virus sequences [8].

Potentially large numbers of new viruses can be discovered from prokaryotic cellular fraction metagenomes leading to marked advances in our knowledge of virus-host interactions. The first crucial step is the identification of viral sequences from the mixture of virus and host sequences. Current tools for identifying virus and provirus sequences have largely taken the same general approach—identifying query sequences with significant similarity to vetted databases of viral sequences (lytic viruses and/or proviruses). Tools for identifying proviruses from within prokaryotic genomes were first developed before the metagenomic era. While identifying proviruses is a different problem than finding viral sequences within mixed metagenomic samples, provirus finding tools laid the groundwork for current approaches and in general use the same principles used in current metagenomic tools. These provirus finding programs include Phage_Finder [9], Prophinder [10], PHAST [11], and PhiSpy [12]. They generally use sliding windows analyses to scan for regions that have high densities of genes with significant similarity to databases of known virus genes and predict those regions as proviruses. Phispy [12] further integrates multiple information sources other than similarity-based methods such as protein length, AT and GC skew, transcription strand direction (long stretches of genes in viral genomes are frequently encoded on the same strand), and unique virus *k*-mers to further increase the detection accuracy. These prophage detectors are not well suited for identifying viral sequences from assembled metagenomic data, because most assembled contigs are short, fragmented contigs and possess few or no complete predicted genes. Additionally, most of these prophage predictors are not optimized to process large numbers of contigs in a reasonable time (see [13]), the exception being PHASTER, an updated version of PHAST [14].

Studies and tools aimed at identifying viral sequences in mixed metagenomic datasets likewise often use similarity searches of reads or assembled contigs to known virus reference genomes. For example, Waller et al. [15] detected 15 virus genera in 252 human gut metagenomic samples by mapping short reads to known phage marker genes, and numerous novel virus-host interactions were subsequently established. The tool VIROME, while built to specifically analyze viral fraction metagenomes, categorizes predicted proteins as microbial or viral via blast searches against metagenomic databases and UniRef proteins [16]. Metavir [17] is a web server tool that can rapidly compare metagenomic reads to complete viral genomes from the Refseq database, and this tool has addressed the issue of processing large amounts of sequence data not implemented in most virus or provirus finding tools. The tools Centrifuge [18] and DIAMOND [19] can rapidly map metagenomic reads to optimally indexed nucleotide or protein databases respectively, and can be useful in identifying viral genes in metagenomics. Such reference-based inferences, however, are hampered by the limited extent to which current reference databases represent the extant diversity of natural viral communities, i.e., this approach only finds viruses closely related to those we already know about. It is estimated that only about 15% of viruses in human gut microbiome and 10% in the ocean have similarity to the known viruses [3, 20].

VirSorter [13] is the most recent and best performing program for detecting viral sequences in assembled metagenomics data. It can detect both proviruses and lytic viruses. Beyond simply identifying regions enriched in genes with similarity to viral sequence databases, it follows in the footsteps of PhiSpy by integrating multiple types of evidence including presence/absence of viral hallmark genes, enrichment of viral-like genes, enrichment of uncharacterized genes, and depletion of Pfam-affiliated genes. VirSorter relies heavily on similarity searches to available viral databases, but it has an added advantage that it uses a manually curated database of virus reference genomes augmented with metagenomic viral (virome) sequences sampled from freshwater, seawater, and human gut, lung and saliva. Another advantage is the use of the strand switching and short gene criteria that are characteristic viral phenomena that do not require similarity searches. VirSorter, however, is still gene-based and requires at least three predicted genes within a contig to make a prediction, thereby excluding many shorter contigs. It is known that some viruses have about 11–14% non-coding regions [21, 22], and this too could hinder gene-based virus prediction programs. In addition, high confidence VirSorter predictions (categories I and II for “most confident” and “likely” predictions) rely on the presence of a hallmark viral structural gene, which again will limit detection of fragmented viral contigs. Similarly, VirSorter may miss many novel viruses for which their hallmark genes have not been characterized or are poorly represented in reference databases, and novel viruses not well represented in existing viromes can also be missed.

Sequence signature methods provide a promising and wholly different new avenue for improved identification of viral contigs. Characterization of sequences using frequencies of *k*-mers (or *k*-tuples, *k*-grams) regardless of coding and non-coding regions, have been used with great success for many sequence discrimination and classification applications. Several *k*-mer based tools, including Glimmer [23], Phymm [24], PhyloPythiaS [25], Kraken [26], CLARK [27], k-SLAM [28] exist for identifying or classifying prokaryotic metagenomic sequences, but they notably do not attempt to discriminate or classify viral sequences. For example, Glimmer uses interpolated Markov models learned from distributions of *k*-mers to identify bacterial genes from mixed samples containing eukaryotic sequences [23], and Phymm taxonomically classifies bacterial sequences as short as 100 bp using the same type of models [24]. PhyloPythiaS is another web server for taxonomic assignment of bacterial sequences that uses an ensemble of machine learning classifiers trained on *k*-mer frequencies [25]. Moreover, Kraken, CLARK, and k-SLAM use *k-*mers to index genomic sequences and speed up sequence comparison for taxonomically assignment of prokaryotic metagenomic sequences [26–28]. In general, sequence signatures-based methods characterize sequences using *k*-mers from the whole sequence, coding or not, and then build a classification model based on the distribution of *k*-mers. Since no gene finding or gene similarity comparisons are needed, these methods can have superior performance for short sequences that have only a few or incomplete genes. The use of discretized *k*-mer patterns also avoids the reliance on hallmark genes or alignment to known viruses. Finally, if viruses universally use some *k*-mer patterns distinct from prokaryotes, then word-based methods may be more powerful in identifying novel viruses that are distantly related to currently known viruses and lack homologous sequences at the gene level.

The motivation for developing a *k*-mer based tool for viral sequence was inspired during development of another tool, VirHostMatcher [29], that matches query viruses to their probable hosts based on *k*-mer frequency similarities. We and others have shown that viruses often share higher similarity in *k*-mer patterns with its specific host than with random hosts [29, 30], presumably because of evolutionary selection to share similar codon usage. VirHostMatcher utilizes this phenomenon to predict the probable host of a query virus by identifying to which host sequence the virus has the greatest *k*-mer similarity (using the d2* measurement [29]). In the process of developing VirHostMatcher, we noted other virus sequences often had greater *k*-mer similarity scores to the query virus than any of the other host sequences. This initially suggested that in addition to specific viruses and hosts sharing *k*-mer patterns, viruses themselves may share some characteristic *k*-mer patterns that could potentially be used to distinguish viral and host sequences.

In this paper, we have developed VirFinder, to our knowledge, the first *k*-mer based program for identifying prokaryotic viral sequences from metagenomic data. VirFinder uses machine learning methods to identify sequence signatures that distinguish viral sequences from host sequences, and as a result, constructs a scoring system to predict viral sequences based on sequence signatures. We have evaluated VirFinder for its performance in detecting viral sequences over a range of short (500 bp) to long ≥ 3000 bp sizes, including novel viruses. VirFinder exhibits improved performance over VirSorter in correctly identifying novel viruses, especially for short (1000 bp) contigs. VirFinder was applied to find and analyze viral sequences in human gut metagenomic data from healthy and liver cirrhosis patients. Diseased patients exhibited lower viral diversity, and 15 viral contig bins could be used to predict the disease status of liver cirrhosis with high statistical power, demonstrating the potential use of metagenomic viral diversity in the diagnosis of human disease states. VirFinder is available at https://github.com/jessieren/VirFinder.

## Results

To build and test VirFinder, two separate sets of viral and host sequences were used for training and testing of the machine learning tool: RefSeq virus and prokaryotic genomes sequenced before 1 January 2014 for model training and after 1 January 2014 for testing. The partitioning of training and evaluation sequences by date was used to evaluate the ability of the tool to discover novel viruses based on patterns of previously known viruses. The 1 January 2014 break point was selected to make fair comparisons in performance to VirSorter, which makes predictions based on a database of viruses sequenced before January 2014. To mimic fragmented metagenomic sequences, RefSeq virus genomes were split into non-overlapping fragments of various lengths *L* = 500, 1000, 3000, 5000, and 10,000 bp, and the same number of non-overlapping fragments were randomly subsampled from the prokaryotic genomes (Table 1). See Methods for details.

**Table 1:** The number of fragments generated from the virus genomes discovered before and after 1 January 2014

Fragment length (*L*)	Before 1 January 2014	After 1 January 2014	Total
500 bp	154,640	50,350	204,990
1000 bp	77,014	25,087	102,101
3000 bp	25,263	8246	33,509
5000 bp	14,881	4878	19,759
10000 bp	7120	2357	9477

### The effects of *k*-mer length and contig length

We first determined the best word length to use with VirFinder and how contig length of both training sequences and query sequences affected prediction performance. A logistic regression model with lasso regularization was chosen for its good interpretability and flexibility. The machine learning model was trained using equal numbers of sequences subsampled from prokaryotes and viral genome sequences at several contig lengths: 500, 1000, 3000, 5000, and 10000 bp. For prediction of each query sequence, VirFinder first extracts the *k*-mer features from the sequence and then generates a score between 0 and 1 based on the trained machine learning model, with a higher score indicting higher possibility that the sequence is viral. The tool also outputs a statistical measure of how distinct it is from prokaryotic hosts contigs: the *p* value from comparing the query score to the distribution of scores for all host contigs used in the training dataset.

### Evaluation of VirFinder with contigs subsampled from known virus and host genomes

**Fig. 1:**
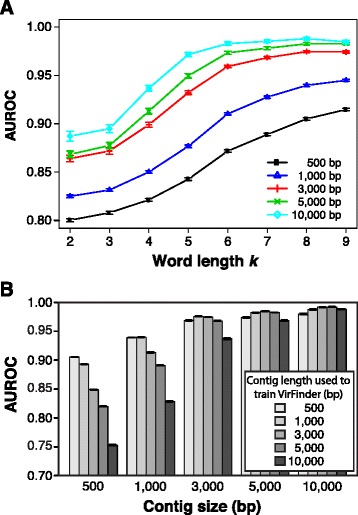
The impact of *k*-mer size and contig length on the performance of VirFinder. VirFinder was trained using contigs sampled from viral and host genomes sequenced before 1 January 2014 and predictions were made on contigs from genomes sequenced after 1 January 2014. Error bars depict standard error determined from 30 bootstrap samples from the testing dataset. **a** Area under the curve for receiver operator curves (*AUROC*) are shown when VirFinder was trained using several *k*-mer lengths and contig lengths. **b** AUROC values for VirFinder results when using *k*-mer length 8 and several combinations of contig lengths used for training and testing

After training the model, VirFinder was evaluated on equal numbers of viral and host contigs subsampled from genomes sequenced after 1 January 2014. To evaluate VirFinder’s performance, we used receiver operating characteristic (ROC) curves typically used to evaluate performance of classifiers. ROC curves were generated by setting a score threshold and calling contigs as viral if their scores were above that threshold. Over a range of incrementally decreasing thresholds (or increasing thresholds for *p* value), we calculated and plotted the fraction of true viral contigs that were correctly called as viral or the true positive rate (TPR) and the fraction of prokaryotic contigs that were incorrectly called as viral or the false positive rate (FPR). The area under the curve of these ROC curves (AUROC) was used to evaluate performance whereby high values indicate good performance. A score of 1 represents perfect identification of all true viral contigs with no false positives, and a score of 0.5 represents a random classification. For VirFinder, AUC values and thus performance increased as *k*-mer length increased (Fig. 1a). For contigs with length ≥ 3,000 bp, performance was relatively stable at *k*-mer lengths ≥ 6. For 1000 bp contigs, AUROC values began to stabilize for *k*-mer size ≥8, and for 500 bp contigs, performance appeared to still increase appreciably above *k*-mer size 8. Based on these results, *k*-mer length 8 was chosen for all subsequent applications of the tool. At *k*-mer length 8, AUROC score first increased somewhat from 0.91 for 500 bp contigs to 0.94 for 1000 bp contigs and then were relatively stable for higher lengths (0.97, 0.98, and 0.99 for 3000, 5000, and 10000 bp contigs). Overall, these high AUROC scores demonstrate the strong ability of our VirFinder tool to correctly identify newly obtained viral sequences.

Metagenomic assembly produces contigs of various lengths ranging from hundreds of bp to 10^5^ bp or more, so we wanted to determine the sensitivity of VirFinder’s performance to different contig lengths used for training and evaluation. As before, the model was trained using contigs subsampled from genomes sequenced before 1 January 2014 and then tested on contigs subsampled from genomes sequenced after 1 January 2014. For a given query contig length, AUROC scores in general were highest when the query contig length matched the contig length on which the model was trained, and performance dropped as the training model contig length was increased or decreased (Fig. 1b). Performance was more sensitive to changes in the training contig length for smaller query contig lengths. In particular, for query contigs ≥ 3000 bp, AUROC scores did not appreciably change across the different training contig lengths. Based on these results, we subsequently used the 500 bp trained model to predict contigs *<*1000 bp; the 1000 bp trained model to predict contigs of 1000–3000 bp; and the 3000 bp model predicts contigs with length ≥ 3000 bp.

Because metagenomic datasets may contain different proportions of viral and host contigs, this potentially could affect prediction performance for a tool that is trained on equal proportions of viral contigs. In practice, the fraction of viral contigs will vary with different types of samples, so VirFinder was evaluated as above using subsampled host and viral contigs sequenced after 1 January 2014, but with 10 and 90% viral mixtures. Within each contig size class, AUROC scores had no obvious differences (≤1.8%) between different fractions of viral mixtures (Additional file 1: Figure S1A). Area under precision-recall curves (AUPRC) were used as a complementary method to evaluate the prediction performance, as they are more sensitive for imbalanced data. In these plots, precision or the fraction of predicted viral contigs that are truly viral is plotted against and recall the fraction of viral contigs that are correctly called (also known as true positive rate (TPR)). As with AUROC scores, they range from 0 to 1 with higher values indicating better performance. For each contig size class tested (as in Fig. 1), AUPRC values increased with increasing fraction of viruses in the contigs tested (Additional file 1: Figure S1B), and these increases were more pronounced for smaller contig sizes. For example, AUPRCs were all nearly 1 (perfect prediction) with samples containing 90% viral contigs regardless of contig length, while they were 0.71, 0.94, and 0.99 for 1000 bp contigs for 10, 50, and 90% viral contig datasets, respectively.

### Comparison of VirFinder and VirSorter performance

**Fig. 2:**
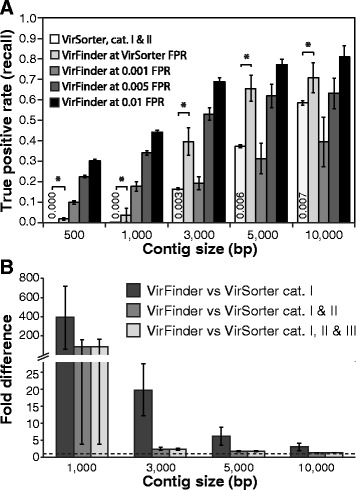
Performance of VirSorter and VirFinder virus prediction for contigs subsampled from virus and prokaryotic genomes. As input to each evaluation, equal numbers of contigs subsampled from virus and prokaryotic genomes were used for 30 replicate bootstrap samples. **a** The fraction of true viral contigs (true prediction rate (TPR)) identified by VirSorter using category I and II predictions and VirFinder at the same false positive rate (*FPR*) as VirSorter (listed in or above the VirSorter bars) and at FPRs of 0.001, 0.005, and 0.01. *Bars* depict mean values for 30 replicate bootstrap samples and *error bars* depict the standard error. *Asterisk* indicates the TPR of VirFinder is significantly higher (*p* < 0.001) than that of VirSorter at the same false positive rate (Wilcoxon signed-rank one-sided test). **b** The ratio between mean VirFinder and VirSorter true positive rates for category I; I and II; and I, II, and III where VirFinder FPRs were set at the corresponding FPRs of VirSorter predictions. Since VirSorter had no predictions for 500 bp contigs (TPR = 0), the ratio is infinite (not shown). *Error bars* depict mean standard error, and the *red line* shows a ratio of 1

We assessed the ability of VirFinder to correctly identify viral contigs in comparison to VirSorter, the top performing viral classification tool [13]. Both VirSorter and VirFinder were tested using the same set of evaluation contigs as above: equal numbers of contigs subsampled from host and virus genomes sequenced after 1 January 2014. This provided a fair comparison as both methods use databases of viruses sequenced before 1 January 2014 to make their predictions. We first assessed the true positive (TPR) and false positive rate (FPR) for VirSorter using category I and II VirSorter predictions. Categories I, II, and III represent “most confident”, “likely”, and “possible” predictions (see [13] for details). In comparing the performance of two methods, it is important to use the same criteria for a fair comparison, namely, comparing the true positive rate under the condition of both methods having equivalent false positive rates. To do this, we first determined the true and false positive rates for VirSorter with category I and II results (or when noted, with different levels). By selecting the VirFinder score threshold that produces the same FPR level as the corresponding VirSorter results, we then determined the true positive rate (TPR) for VirFinder. At all query contig lengths, VirFinder’s TPR exceeded that of VirSorter (Fig. 2a) at comparable FPRs, and based on 30 replicate bootstrap samples, this difference was statistically significant (*p* ≤ 6 × 10^−5^, Wilcoxon signed-rank one-sided test). The relatively large standard errors of TPRs were primarily due to the large range of FPRs from VirSorter’s results and thus the subsequent determinations of VirFinder’s TPRs. Note that for 500 bp contigs, VirSorter made no predictions so its FPR and TPR were both 0. VirFinder correctly identified 944 contigs as viral (TPR = 1.9%) at 0 FPR in this case. VirFinder correctly identified 78, 2.4, 1.8, and 1.2 times more viral contigs than VirSorter for 1000, 3000, 5000, and 10000 bp, respectively (Fig. 2b), and therefore VirFinder had a substantially larger TPR for 1000 bp or shorter contigs. TPRs for VirFinder were also evaluated at three specific fixed FPRs. At FPRs of 0.01 and 0.005, VirFinder had higher TPRs at all contig lengths than VirSorter. At the more conservative FPR of 0.001, VirFinder had TPRs 18, 19, 31, and 39% for 1000, 3000, 5000, and 10000 bp contigs, respectively.

Along with the tests above on equal numbers of virus and host contigs, we also tested VirFinder and VirSorter using highly skewed contig datasets: host-enriched (10% viral) and virus-enriched (90% viral). At all contig lengths and both viral fractions, VirFinder’s TPR exceeded that of VirSorter (Additional file 1: Figure S2A, S2B). For example, for 1000 bp contigs, VirFinder predicted 1.2, 3.6, and 11% of true viral contigs while VirSorter predicted 0.04, 0.05, and 0.26% for 10, 50, and 90% viral samples, respectively. This translates to 26-, 78-, and 41-fold higher TPRs for VirSorter. For long contigs >3000 bp, the fold difference in VirFinder over VirSorter TPRs were lower, on average 1.1, 1.8, and 3.8 for 10, 50, and 90% viral samples, respectively.

### Sensitivity of VirFinder to mutations

Because VirFinder relies on nucleotide *k*-mer analyses and sequencing technologies can contain errors, the sensitive of VirFinder to mutations was tested. This also served as a test of the ability of VirFinder to identify viruses that may rapidly diverge over time from previously sequenced virus strains in the training datasets due to high mutation rates in viruses. Random mutations were introduced into the 30 replicate subsampled contigs used above in the analyses in Fig. 2 at three different rates (0.0001, 0.001, and 0.01), and AUROC values were compared to the results with no mutations. Within each contig size group, only at the highest rate of 0.01 mutations per bp were AUROC values significantly lower (*p* < 0.01, *t* test) (Additional file 1: Figure S3). VirFinder’s ability to correctly identify viral contigs is insensitive to virus mutation rates of ≤0.001 as suggested in [31]. The reported rates for sequencing errors generated by the Illumina platform are about 0.001 [32].

### Virus prediction for sequences from different host domains and phyla

**Fig. 3:**
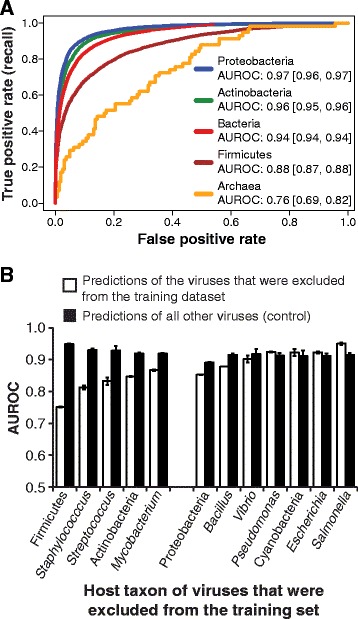
Differences in VirFinder’s performance for different groups of viruses and when excluding particular viruses from the training of VirFinder **a** ROC curves for VirFinder prediction results based on contigs subsampled from viruses isolated on particular host domains and phyla. *Curves* depict mean results for 30 replicate samples each. *Numbers* in the legend indicate mean AUROC values and *numbers* in brackets indicate the upper 2.5% quantiles for 30 replicate bootstrap samples. **b** Viruses that infect four major phyla and eight major genera of hosts were each excluded from the dataset of sequences used to train VirFinder. AUROC scores were then determined when making VirFinder predictions on contigs of the excluded viruses when they were mixed with equal numbers of contigs of other viruses and equal numbers of host contigs as the total number of viral contigs. As a control, AUROC scores were compared to results of predictions of all other viruses. Contigs for the training and evaluating datasets were sampled at a length of 1000 bp, and predictions were made for 30 replicate datasets for each taxon analysis

Recognizing the unevenness in the taxonomic diversity of hosts from which the training set of viruses were isolated, we evaluated how the performance of our tool varied for several groups of viruses. The model was trained on all viruses in the training set regardless of host taxonomy, and AUROC curves were plotted for identification of viruses that infect different domains and major bacterial phyla using 1000 bp subsampled contigs. AUROC scores were markedly lower for archaeal viruses versus bacterial viruses (Fig. 3a). Only 3% of viruses in the training set are archaeal. Similarly, identification of Firmicutes viruses had a notably lower AUROC score, 0.88, than those for Proteobacteria (0.97) and Actinobacteria (0.96). The patterns for other contig lengths were consistent with results shown for 1000 bp contigs (data not shown).

### Assessment of the identification of novel viruses

To assess the ability of VirFinder and VirSorter to identify novel viruses, predictions were also made on whole virus genomes sequenced after 1 January 2014 (*n* = 337), and we paid particular attention to 45 viruses (13%) that had no significant nucleotide similarity (blastn search, *E* value <10–5) to previously viral genome sequences, which we used as a proxy for what we refer to as novel viruses. Using a *p* value cutoff of 0.01 for VirFinder predictions and category I and II VirSorter predictions, VirFinder and VirSorter both predicted most of the viruses (*n* = 26, 58%), and 3 were not predicted by either (Additional file 1: Table S1). VirFinder uniquely predicted 4 viruses that VirSorter did not, and VirSorter correctly predicted 12 viruses that VirFinder did not.

We also assessed the ability of VirFinder to identify novel viruses by excluding viruses that infect a particular group of hosts (four major phyla and eight major genera) and the hosts of that taxon group from the training dataset and then predicting those viruses from among mixed sets of subsampled virus and host contigs. As above, predictions were made on 30 replicate datasets with equal numbers of total host and viral contigs. Prediction results of the selected viruses that were excluded from the training set were assessed with AUROC values and compared to control results of predictions made for all other viral contigs using that same trained version of VirFinder (Fig. 3b). AUROC scores for predicting viruses infecting Firmicutes, *Staphylococcus*, *Streptococcus*, Actinobacteria, and *Mycobacterium* decreased by >5% when they were excluded from the training dataset. Viruses infecting other taxa such as Proteobacteria, Cyanobacteria, and *Escherichia* had reduction in AUROC of <5%, suggesting that they can be predicted reasonably well even when they were excluded from the training data.

### VirFinder’s performance on assembled contigs from simulated metagenomic samples

**Fig. 4:**
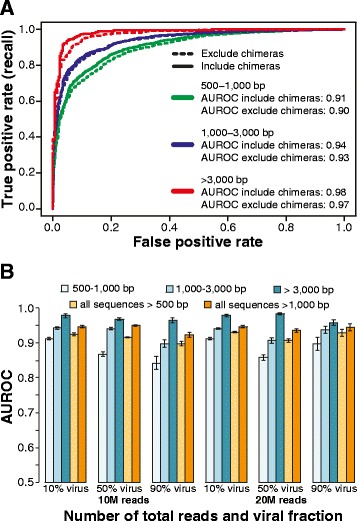
Results for VirFinder predictions made on contigs assembled from simulated human gut metagenomes. **a** The ROC curves and AUROC scores for results from 3 size classes of assembled contigs (20 million read simulated metagnome, 10% viral community). **b** The effects of sequencing depth, viral fraction, and contig length on prediction performance. *Bars* shown mean AUROC scores and *error bars* depict standard error evaluated for 30 bootstrap samples

To evaluate performance on a more realistic dataset, VirFinder and VirSorter were evaluated on contigs assembled from simulated human gut metagenomic samples. A simulated human gut metagenome with 20 million reads was generated using NeSSM [33] by subsampling reads from host and viral reference genomes found in a real Human Microbiome Project gut metagenomic sample at their respective relative abundances (see Methods). Assembly with metaSPAdes [34, 35] generated 190,079 contigs ≥500 bp in length, and each contig was definitively assigned as prokaryotic (88%), viral (10%), or ambiguously chimeric (1.8%) (see Methods). VirFinder’s performance was first assessed using AUROC values as before for contigs 500–1000 bp, 1000–3,000 bp, and ≥3000 bp in length. Viral contigs from genomes sequenced after 1 January 2014 paired with the same number of randomly sampled host contigs were evaluated. AUROC scores were all very high, *>*0.9, and increased with increasing contig length. For 1000–3000 and ≥3000 bp contigs, the AUROC scores were as high as 0.94 and 0.98, respectively (Fig. 4a). Including chimeric contigs decreased performance slightly. To study the effects of sequencing depth and the fraction of viral sequences in the sample, simulated metagenomic samples were generated for 10 and 20 million total reads and using 3 different viral and host proportions (10, 50, and 90% viral reads, see Methods). The AUROC scores based on 10 and 20 million reads are similar, indicating that within the sequence depth levels investigated, sequencing depth does not markedly affect the viral contig prediction results (Fig. 4b). As before, AUROC values generally increased noticeably with contig length, but within size classes, they did not appreciably differ between 10, 50, and 90% viral samples tested (<8% for 500–1000 bp and <5% for all other size classes). To make fair comparisons across scores predicted using different models trained using contigs of different lengths, VirFinder *p* values rather than VirFinder scores were used for generating ROC curves and corresponding AUROC scores. The AUROC scores using *p* values were 0.90–0.93 for contig *>*500 bp and 0.92–0.95 for contig *>*1000 bp. AUPRC results showed increasing values with increased proportion of viral contigs present in the sequences being tested (Additional file 1: Figure S4).

**Fig. 5:**
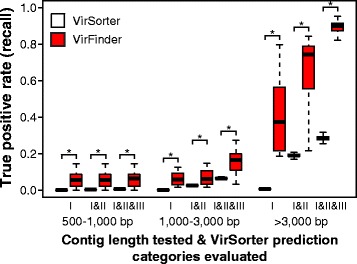
Evaluation of VirFinder (VF) and VirSorter (VS) predictions on contigs for 3 length ranges assembled from 20 M reads simulated human gut metagenomes at 50% viral level. *Bars* depict true prediction rates (TPRs) for VirSorter category I; I and II; and I, II, and III predictions. As in Fig. 2, VirFinder predictions were evaluated at the same false positive rates (FPR) as corresponding VirSorter results. Thirty replicate bootstrap samples of contigs assembled from simulated metagenomes were tested for each condition. Metagenomes were simulated based on the relative abundance of complete virus and host genomes found in a real human gut metagenome. The *horizontal bar* displays the median, *boxes* display the first and third quartiles, and *whiskers* depict minimum and maximum values. *Asterisk* indicates VirFinder’s TPRs are significantly larger than VirSorter’s (Wilcoxon signed-rank one-sided test, *p* < 10^−4^)

VirFinder and VirSorter’s TPRs were next compared using assembled contigs from simulated 20 million read metagenomes. TPRs were compared at the same FPRs levels as found for VirSorter considering various VirSorter category results (I, “most confident; II, “likely”, III, “possible”). VirFinder correctly identified more viral contigs than VirSorter at all ranges of contig lengths and for all three combinations of VirSorter categories used when tested with samples with equal numbers of viral and host contigs (Fig. 5a). As before, use of 30 replicate bootstrap samples showed that these differences were statistically significant (*p* ≤ 10^−5^, Wilcoxon signed-rank one-sided test). VirSorter identified almost no viral contigs of 500–1000 bp viral contigs at the 3 category combinations used (I, I and II, and I–III) (TPRs were 0, 0.2, and 0.5%, respectively). At the same FPRs levels as VirSorter, VirFinder successfully identified 5.9, 5.9, and 6.0% true 500–1000 bp viral contigs for the 3 VirSorter category combinations, respectively, which are undefined (VirSorter TPR was 0%), 30 and 13 times higher than VirSorter’s TPRs. For contig lengths 1000–3000 and >3000 bp, both methods had higher TPRs. VirSorter’s TPRs were 2.5 and 19% for 1000–3000 and >3000 bp, respectively, using categories I and II. At the same controlled FPR, VirFinder’s had TPRs of 7.4 and 67%, or 3.0 and 3.5 times higher than VirSorter’s TPRs.

Viral prediction performance was also evaluated using unbalanced fractions of viral and host contigs. Overall, VirFinder’s TPRs were significantly higher than VirSorter’s TPRs for all 3 ranges of contig length, all 3 VirSorter category combinations considered, and all datasets with different viral fractions (*p* ≤ 10–3) (Additional file 1: Figure S5). Performances were also evaluated when parsing the results for all contigs >500 bp or all contigs >1000 bp. The simulated assembled contig dataset contained on average 41% 500–1000 bp contigs, 38% 1000–3000 bp contigs, and 20% >3000 bp contigs. When considering all contigs >500 bp, VirFinder had significantly greater TPRs than VirSorter except when comparing category I or category I and II VirSorter results with 10% viral samples and when comparing category I and II VirSorter results with 50% viral samples (Additional file 1: Figure S6). When considering all contigs >1000 bp, however, VirFinder had significantly higher TPRs than VirSorter under all scenarios at equivalent FPRs (Additional file 1: Figure S6).

### Example application: identification and analysis of viral communities in human gut metagenomes from a liver cirrhosis study

We applied the VirFinder tool to study real human gut metagenomes of healthy and liver cirrhosis patients. Qin et al. [36] previously reported the alteration of human gut microbiomes associated with liver cirrhosis. Their analysis only focused on the bacterial microbiome by mapping reads to reference bacterial genomes. Roughly 40% of the reads were unaligned, indicating that the remaining reads represent unknown bacterial or archaea or more importantly prokaryotic viruses. We reanalyzed the Qin et al. dataset using VirFinder (and VirSorter) to identify viruses in these metagenomes and any potential differences in the prokaryotic viromes of healthy and diseased patients.

Reads from 40 healthy and 38 liver cirrhosis patients, comprising 316 Gb of total sequence data were cross-assembled. Only the resulting 325,020 contigs that were >1000 bp in length were retained to achieve high prediction accuracy. The majority (76%) of contigs were 1000–3000 bp long (Additional file 1: Figure S7). VirSorter predicted 2657 contigs as viral using category levels I and II. To make fair comparisons, we also analyzed the 2657 contigs with the highest VirFinder scores. The false positive rate for these VirFinder contigs was estimated at 15% using *q* values estimated by the positive false discovery rate (pFDR) method [37, 38].

**Fig. 6:**
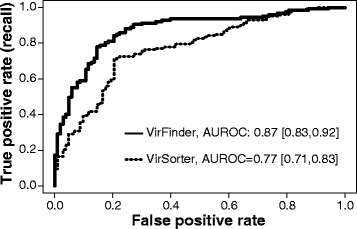
ROC curves and AUROC scores for classifying healthy and liver cirrhosis patients based on abundance profiles of predicted viral contig bins. The models were trained using averaged bin coverage (averaged RPKM of contigs in the bin) of 78 samples of health and diseased patients and then tested on an independent set of 230 samples from the same study. Each set of 2657 viral contigs predicted by VirFinder and VirSorter were separately binned based on sequence tetranucleotide frequencies and contig coverages normalized by contig length and number of mapped reads in samples. The *numbers* in the square brackets are the lower and upper 2.5% quantiles

Contigs were binned using COCACOLA [39] based on *k*-mer frequencies and abundance patterns across samples to group similar contigs. This produced 86 and 116 bins for VirSorter and VirFinder contigs, respectively. The abundance profiles of contig bins across 78 samples of health and diseased patients were used to train classification models to distinguish health status. The logistic regression model with lasso regularization was used in order to enhance the prediction accuracy and interpretability. These models were then tested on an independent set of 230 samples from the same study [36] using ROC curves and AUROC scores. Model results using VirFinder binned contigs had a larger AUROC score, 0.87, than those for VirSorter bins, 0.77. The ROC curve for VirFinder was above that of VirSorter for most of the plot (Fig. 6). The high AUROC scores demonstrate that virome data mined by virus prediction software can predict with good confidence the health status of patients. The ROC results furthermore indicate that viral contigs predicted by VirFinder can better distinguish between healthy individuals and liver cirrhosis patients.

Similarity searches of contigs and their predicted proteins against NCBI’s non-redundant nucleotide (nt) or protein (nr) databases were conducted to determine if contigs and bins do not have any similarity to previously known sequences and thus potentially represent novel viral sequences. Searches against nt and nr assessed if contigs are closely or distantly related to known sequences, respectively. Only 12% of contigs were ≥95% similar to another bacterial or viral sequence in the nt database (requiring an *E* value <1e^−10^ for ≥100 bp). It has recently been suggested that virus species distinguish from each other by ~95% nucleotide identity [40] thus the majority of the contigs probably represent new viral species. Analyzed in another way, 64% of contigs did not have any significant blastn matches to the nt database (*E* value <1e^−10^ for ≥100 bp), such that 17% of the bins had no contigs with any significant similarity to nucleotide sequences in this database. For blastp searches, only 8% of contigs did not contain any predicted encoded protein with significant similarity (*E* value <1e^−5^, bit score ≥ 50) to any protein in the nr database. This translated to all but two bins that had at least one protein with significant similarity to a previously reported protein sequences. These 2 bins were comprised of 1 short contig each, 1119 and 1182 bp, with 1 and 3 predicted proteins on those contigs, respectively. We also note that, of 2657 contigs, ~10% had at least 1 protein for which its best hit to nr was a viral protein or has significant similarity to a viral Pfram domain. This resulted in 57% of bins having at least 1 viral protein, supporting that these binned contigs indeed are viral sequences.

**Fig. 7:**
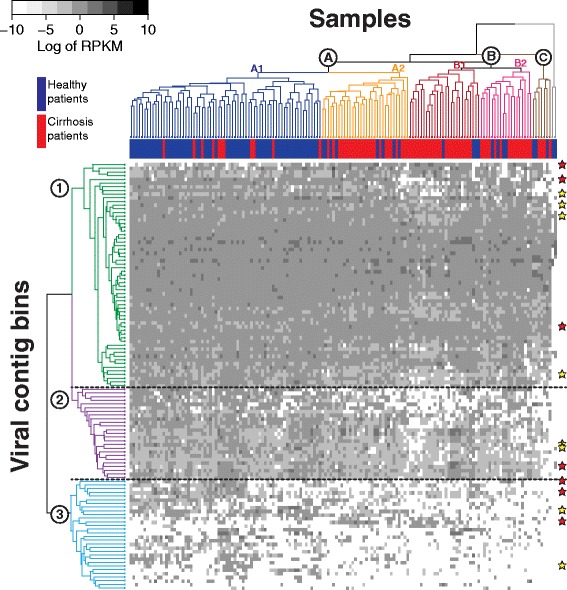
Two-way hierarchical clustering of viral contig bins and human gut metagenomic samples from a liver cirrhosis study [36]. Rows are contig bins (*n* = 116) comprised of 2657 viral contigs predicted by VirFinder, and columns are samples from healthy (*blue*) and cirrhosis (*red*) patients (*n* = 80 and 76, respectively). The *heatmap* depicts bin coverage across samples calculated as the averaged Reads Per Kilobase per Millions mapped reads (RPKM) of contigs in each bin. Bins form three coherent clusters that generally correspond to bins that are found in nearly all (95% on average), most (82% on average), or some (33% on average) samples. With the exception of two outliers (in *grey*), samples belong to three major clusters, A, B, and C, and clusters A and B each have two coherent subgroups (A1, A2, B1, B2). *Stars* depict bins that are positively (*red*) or negatively (*yellow*) associated with cirrhosis samples chosen using the lasso method for subset variable selection

**Fig. 8:**
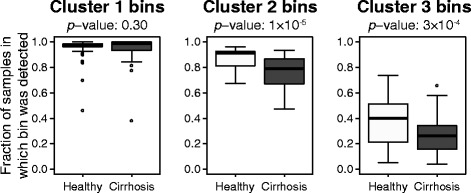
Box plots summarizing the number of healthy or cirrhosis patient samples in each viral bin. Results are summarized from the three bin clusters shown in Fig. 7. *Horizontal bars* indicate median values, *boxes* depict the first and third quartiles, *whiskers* depict minimum and maximum values, and outliers are shown as points. *p* values for Wilcoxon signed-rank one-sided tests comparing results for healthy and cirrhosis samples are listed above each graph

Hierarchical clustering was used to cluster contig bins and patient samples (80 from healthy and 76 from cirrhosis patients) according to the relative abundance of bins (Fig. 7). Viral bins (rows) formed three major groups broadly reflecting the degree to which the bin was present across samples. Bins belonging to cluster 1 (green), 2 (purple), and 3 (blue) were found, respectively, in nearly all samples (95% of samples on average), most samples (82% on average), and only some samples (33% on average), respectively. For bins in clusters 2 and 3, their prevalence in cirrhosis patient samples was significantly lower than those of healthy patients, 76 vs. 88% and 27 vs. 39%, respectively (Wilcoxon signed-rank one-sided test, *p <* 0.001), while there was no difference for cluster 1 (95 vs. 96%, *p* = 0.30) (Fig. 8). Overall fewer bins were detected in diseased than healthy patients (89 vs. 93% bins detected on average, respectively, *p* < 10^−4^).

Except for two outlier samples (in grey) for which few bins were detected, samples clustered into three major groups (A, B, and C), and the A and B groups contained two coherent subgroups each (A1, A2, B1, and B2; Fig. 7). The A1 subgroup (in blue) contained 70 samples that were comprised mostly of healthy patient samples, while samples from cirrhosis patients primarily fell into the other subgroups. Among the samples in all groups except for the 2 outliers, 10 healthy and 14 cirrhosis patients had multiple (>1) samples. The Pearson correlation coefficients between contig coverages (Reads Per Kilobase per Million mapped reads (RPKMs)) of viral bins of samples within those individuals were significantly higher than that between samples from different individuals (Wilcoxon rank-sum one-sided test, *p* < 10^−4^), even when evaluated separately using the 3 different groups of viral bins.

Using the lasso regularization method for variable selection [41, 42], we identified 7 and 8 bins that were positively or negatively associated with the disease status, respectively (Table 2). Nine of these bins had at least one protein for which its best blastp search results against nr was a viral protein or the protein had significant similarity to a viral Pfam domain. Of particular note were bins 2 and 64 that were negatively associated with cirrhosis and had 8 and 19 proteins, respectively, that from blastp searches against nr were most similar to crAssphage proteins. Bin 64 in particular contains 2 large contigs (10 and 12 kb) that have several predicted proteins with similarity to (26 to 66% protein identity) and in similar order to large regions of the crAssphage genome (Additional file 1: Figure S9). Also of note is bin 41; the bin most strongly associated with cirrhosis (Table 2), that contains a large 121 kb circular contig. Nearly all (95%) of the prediction proteins on this contig have significant similarity (78% identity on average) to megaplasmid pMP1046B from the Firmicutes strain *Lactobacillus salivarius* strain JCM 1046, representing 91% of the proteins on pMP1046B. This bacterium was isolated from swine intestines. The 121 kb circular contig was only predicted at category level III by VirSorter. Several of these predicted proteins have similarity to Pfam motifs for phage proteins (phage integrases, phage tail tape proteins, phage holins), suggesting that it is a lysogenic phage that is stably maintained in the host as a replicating plasmid like P1 phage [43]. Four other large (40–72 kb) circular contigs were among the 2657 VirFinder predicted viral contigs, and each have several proteins with similarity to phage related Pfams. Bin 41 also contains a 21.6 kb contig that possesses several structural phage genes (capsid, heal-tail adapter genes). Twenty nine of the 32 predicted proteins on this contig, including the structural phage proteins, are most similar to sequences of the genus *Veillonella*, strongly suggesting that this viral contig occurs as a prophage in this genus. A recent analysis of the Qin et al. dataset also found that host sequences of the genus *Veillonella* were more abundant in cirrhosis patients [28], consistent with the higher association of this viral bin with individuals with cirrhosis.

**Table 2:** Summary information for 15 viral contig bins associated with cirrhosis (+) or healthy (−) patients samples

Bin	Coefficients of association with cirrhosis^a^	No. of contigs in bin	Total nucleotides in bin (bp)	No. of predicted proteins in bin	No. of contigs with significant blastn hit to *nt* ^b^	Bin contains proteins with similarity to viral proteins^c^
2	−0.04	46	82431	92	3	Y
6	0.06	88	295063	357	2	Y
35	0.00	1	1214	2	1	N
41	0.23	40	259266	360	15	Y
48	0.05	3	4940	5	0	N
51	−0.19	36	84134	112	6	Y
59	−0.10	68	184455	245	3	Y
64	−0.05	29	130154	148	1	Y
66	0.12	6	8500	7	5	N
69	0.00	1	1197	1	0	N
72	−0.05	29	77421	110	6	Y
78	−0.05	21	43329	48	1	Y
93	0.03	1	1295	1	0	N
106	−0.06	2	5243	7	0	N
127	0.01	18	72694	110	0	Y

^a^Coefficients determined by the logistic regression with lasso regularization method for variable selection (see Methods)

^b^Contig had at least one blastn hit to NCBI’s non-redundant nucleotide database (nt) with an *E* value of ≤1e-10 and an alignment length of ≥100 bp

^c^Bin contains at least one protein for which its best blastp search results against NCBI’s non-redundant protein database (nr) was a viral protein or the protein had significant similarity to a viral Pfam domain (see Methods). Similarity requirements: *E* value of ≤1e-5, bit score ≥ 50

## Discussion

We have presented the development, validation, and application of VirFinder, a machine learning based tool that uses *k*-mer frequencies to accurately identify viral contigs. To our knowledge, VirFinder represents the first virus identification tool that solely uses a nucleotide *k*-mer frequency based approach and stands in contrast to previous methods that primarily use similarity searches of predicted genes to known viral sequences (e.g., Phispy, Phymm, VirSorter). We do note that PhiSpy uses AT and GC skew, a coarse form of *k*-mer analysis, for prediction, but this is one of several search criteria PhiSpy uses. VirFinder adds to the growing number of recently developed tools that use powerful, alignment-free *k*-mer frequency approaches to rapidly categorize and/or analyze the similarity of sequence datasets [24–29, 39, 44–49]. We note here that VirFinder has been trained to identify prokaryotic viruses, but in the future, it potentially could be utilized to identify eukaryotic viruses as well. We note that users applying VirFinder to eukaryotic host associated microbiomes should first filtered out eukaryotic host sequences as VirFinder may potentially misidentify those sequences as viral, since eukaryotic sequences were not included in VirFinder’s training datasets.

VirFinder is trained using a logistic regression model with lasso regularization in order to enhance the prediction accuracy and interpretability. Other regularization methods such as ridge and elastic net had similar performance with no significant difference. We also tested a few other machine learning methods before selecting the logistic regression approach. Performance with a Naïve Bayes classifier was worse than that of logistic regression (data not shown), possibly because of the violation of the independence assumptions for *k-*mer patterns. We also attempted to use the support vector machine (SVM) with kernels, one of the most widely used non-linear classification methods, but it failed because the training sample size was too large (>300,000 sequences) for the regular SVM to handle.

In side-by-side comparisons, VirFinder exhibited superior performance in terms of identifying true viral contigs when evaluated at the same false discovery rates as VirSorter, the current state-of-the-art, gene-based virus prediction tool. VirFinder in particular exhibited much better performance in virus identification for shorter contigs in part because VirSorter is limited to making predictions on contigs that have at least three coding genes. VirFinder’s success is also probably due to its use of *k*-mer frequencies that allows it to extract predictive sequence informative without requiring the presence of specific hallmark genes on the query contig. VirFinder also provides a statistical framework to evaluate if the output score is significantly different from the distribution of host contig scores, which in turn can be used to estimate the false positive rate when selecting a particular VirFinder score threshold (the *q* value approach). Similarly, an additional benefit of our study is that we provide estimates of the false positive rates for VirSorter for contigs of various sizes, which was not extensively evaluated previously. Finally, VirFinder generates prediction scores that are static for each contig regardless of the other contigs being tested at the same time. VirSorter results in contrast are influenced by the particular collection of sequences being analyzed. This is because VirSorter first uses all contigs to estimate the background distributions for the various metrics it evaluates (for example, enrichment of hypothetical genes), and virus predictions are made by comparing individual contig values to these background distributions. Therefore, VirSorter results of individual contigs are not stable and are dependent on what other contigs are included in the query dataset.

VirFinder’s reliance on *k*-mer patterns also gives it a practical advantage over other gene-based methods when analyzing binned contigs. Recent binning methods have advanced analysis of assembled metagenomic contigs by grouping together fragmented contigs belonging to the same or closely related organisms with high fidelity based on tetramer frequencies and abundance patterns across multiple samples [39, 49, 50]. Here, we have used the binning approach COCACOLA that exhibits improved binning accuracy with reduced computational times. Although we have not implemented this here, VirFinder could be applied to binned contigs. Since each bin collectively contains more sequence data than the individual contigs within them, this would presumably result in more robust predictions. Other current methods only make predictions for individual contigs and are not implemented to handle predictions for binned contigs.

VirFinder works by training a machine learning model on known viral and non-viral (prokaryotic host) sequences to detect the additive effect of many subtle differences in the frequencies of *k*-mers specifically used by viruses. A helpful analogy is that viruses and hosts use the same “language”; the genetic code of nucleic acids, but the two have slightly different “accents” or “dialects” in their use of that code, which VirFinder is able to detect. While VirFinder clearly works well in practice, it will be interesting to understand in more detail, the evolutionary phenomena underpinning its success. VirFinder works in principle due to the reasonable underlying assumption that viruses and hosts use different *k*-mer patterns. While there were no individual *k*-mers identified that are unique to viruses or hosts, VirFinder’s machine learning model instead uses the cumulative effect of slight differences in frequencies over many *k*-mers to distinguish virus and host sequences, and the cumulative frequencies of these *k*-mers are significantly different between viruses and hosts (Additional file 1: Figure S8). Viruses and their hosts are very different biological entities, so the different evolutionary selective forces they experience likely shape the *k*-mer space they explore. We hypothesize that evolutionary constraints in particular on capsid structural genes that are unique to viruses may impart some of the *k*-mer signal that allows VirFinder to distinguish virus and host sequences.

In future studies, it will be interesting to determine if there are particular *k*-mer patterns that are universally shared across all viruses, or if it is more the case that distinct patterns exist for each larger group of viruses. The former appears to be the case at a broad level, as viruses exhibit slightly lower GC contents than host genomes [51]. We likewise find that the informative *k*-mers for predicting viruses are significantly more AT-rich (*p* < 10^−4^). It was previously hypothesized that the AT shift was due to the limited availability of G and C nucleotides and higher energetic costs imposed on viruses for utilizing these bases. Further examination of any discernable pattern among the informative *k*-mers could be instructive in understanding the evolutionary and mechanistic reasons for how and why viruses and hosts use *k*-mer space differently.

If the model that viruses universally share certain *k*-mer usage patterns is true, this suggests that VirFinder could have a strong advantage in extending viral prediction to novel virus groups for which we have no sequences. This model is partly supported by results obtained when particular viruses were excluded from the training dataset (Fig. 3b). Most viruses showed differences of <10% in prediction performance as compared to controls, suggesting that VirFinder can readily predict them based on *k*-mer patterns present in the other viruses that were not excluded. While prediction performances for viruses infecting Firmicutes and *Staphylococcus* were 21 and 13% lower than controls, AUROC scores were still generally high, often >0.75. We also found that VirFinder could correctly predict many recently sequenced, novel viruses that lack any significant nucleotide similarity to previously sequenced viruses in the training dataset. Likewise, a significant portion of predicted viral contigs (~30%) from the cirrhosis study have no significant nucleotide or protein similarity to known sequences, and the cirrhosis study recovered several contigs that appear to be variants of crAssphage that were not predicted by VirSorter (Additional file 1: Figure S8).

If instead it is more the case that large subgroups of viruses each have different distinguishing patterns, VirFinder like other virus prediction methods will still be sensitive to the diversity of known viruses represented in the training sequence database. This may explain in part why VirFinder had somewhat lower performance for archaeal viral contigs for which there are relatively few sequenced representatives. Interestingly, VirFinder similarly had somewhat lower performance in identifying Firmicutes viruses compared to other groups even though there were many Firmicutes viruses in the training set. It is unclear why this is exactly the case. As discussed above, the exclusion of certain groups of viruses from the training set did diminish prediction results, suggesting that the viruses that infect particular host taxa do have some signal of *k*-mer patterns that are specific to that group and not shared “universally” (Fig. 3b). We anticipate that VirFinder’s accuracy will improve over time as additional virus isolates are sequenced, especially for archaeal viruses, and available to be added to the training sequence database. To this end, VirFinder can be periodically updated by training it on new, available sequences, and the current release allows users to select and train VirFinder on host and viral databases of their choosing.

One potential counterpoint to the paradigm that viruses and host have distinct *k*-mer patterns is that co-evolution of viruses and their hosts leads to sharing somewhat similar *k*-mer patterns. This is likely due to the evolutionary pressure on viruses to adopt similar codons used by their hosts since they are dependent on host machinery for replication [6, 30, 52–55]. In fact, we and others have previously utilized this phenomenon to predict the probable host of query virus sequences [6, 29, 30]. These two phenomena, virus-host *k*-mer amelioration and viruses and hosts possessing distinguishing *k*-mer patterns, however, may not be mutually exclusive, as some specific distinct viral *k*-mer patterns could still be maintained even in the midst of co-evolutionary pressures to share similar codon patterns with their hosts. In support of this, results obtained during testing of the host-matching tool VirHostMatcher, as discussed in the introduction, indicated that virus-host pairs share some *k*-mer similarity but viruses often appear to share even higher similarity with each other. This supports a model that viruses simultaneously possess distinguishing viral *k*-mer patterns and patterns of virus-host amelioration.

An important consideration in the application of VirFinder is the potential problem of provirus sequences within the host genome training sets and host genes that are present in the genomes of viruses. These “contaminating” sequences in the training set could be expected to potentially reduce TPRs and elevate FPRs, respectively, of virus prediction results. For the case of proviruses, however, we found negligible difference in prediction performance when training VirFinder on a smaller 14,722 host genome dataset [6] vs. training VirFinder on that same database with VirSorter-predicted provirus sequences removed (Additional file 1: Figure S10). This is likely because proviruses only comprise a small proportion, 0.64%, of those prokaryotic genomes. We predict similar, limited impact on proviruses present in our larger host genome database, assuming the fraction of proviruses is similar. Congruent with this, prediction performance as assessed by AUROC values was diminished by <0.5% when random virus isolate contigs were spiked into our host training dataset at 5% (~8-fold larger amount of viruses than found in the Roux et al. dataset) (Additional file 1: Figure S11). Moving forward, we are currently working on “cleaning” our larger host genome database of provirus sequences using VirSorter for a future, updated release of VirFinder.

The presence of host genes in the viral training set is potentially more problematic as this is predicted to inflate the FPR for viral prediction. False positives (true host contigs that are called as viral) should not be an issue for long contigs, but short contigs could potentially be called as viral if they contain a host gene that occurs as a horizontally transferred host gene in the viral training set. A prime example of such genes are auxiliary metabolic genes (AMGs) encoded in viruses that are acquired from their hosts and used to bolster the metabolism of the host for increased virion production [56–58]. The best-studied example of an AMG is the photosynthesis gene, *psbA*, that is encoded in many cyanophage, viruses that infect cyanobacteria [59–61]. Host and viral versions of *psbA* form distinct phylogenetic clusters [62] and have different GC contents and codon usage pattern [63], suggesting that *k*-mer analysis may readily distinguish viral and host *psbA* genes. Using the set of host and viral *psbA* genes from [62], VirFinder correctly predicted 62% of the viral *psbA* genes with no false positives on (using a *p* value cutoff of 0.05). The process of “cleaning” such host genes from viral genomes is not trivial, but based on the results above, host genes in viral genomes likely do not significantly impact VirFinder’s performance.

It was observed that the balance of viral and host contigs can impact the magnitude of prediction performance. The reason for these differences can be explained using the simple Bayes rule. Suppose a contig is randomly picked from the testing dataset. Let *Z* = 1 if the contig is predicted as virus. Let the probability that the contig is a true viral sequence be *P*(V|*Z* = 1), where *V* denotes viruses and *H* denotes hosts. By the Bayes rule,

$$ P\left( V\Big| Z=1\right)=\frac{P\left( Z=1\Big| V\right) P\left(\mathrm{V}\right)}{P\left( Z=1\Big|\mathrm{V}\right) P(V)+ P\left( Z=1\Big| H\right) P(H)}=\frac{P\left( Z=1\Big| V\right)}{P\left( Z=1\Big| V\right)+ P\left( Z=1\Big| H\right)\frac{P(H)}{P(V)}}. $$

where *P*(*V*) and *P*(*H*) are the fractions of viral and host contigs, respectively. Here, *P*(*Z* = 1|*V*) is the probability that a virus can be correctly predicted and *P*(*Z* = 1|*H*) is the probability that a host is falsely predicted as virus. Both *P*(*Z* = 1|*V*) and *P*(*Z* = 1|*H*) do not depend on the ratio $\frac{P(H)}{P(V)}$. As viral fraction *P*(*V*) increases, the ratio $\frac{P(H)}{P(V)}$ decreases, and the probability of having a correct prediction *P*(*V*|*Z* = 1) increases. In fact, *P*(*V*|*Z* = 1) is equivalent to precision, the fraction of predicted viral contigs that are true [64]. Therefore, AUPRC increases as viral fraction increases. For example, for 1000 bp contigs, the AUPRC of VirFinder were 0.71, 0.94, and 0.99 as viral fraction increases from 10, 50, and 90%. Despite these trends, VirFinder still exhibit higher TPRs than VirSorter under almost all conditions tested, the exception being a few cases when considering predictions on all contigs >500 bp (Additional file 1: Figure S6).

While VirFinder shows better performance than VirSorter, we ultimately advocate for the development and use of future tools that combine the core principles of each tool, *k*-mer and gene-based approaches, to make even better predictions. Case in point is the prediction of recently sequenced, novel virus genomes, whereby each method could uniquely identify virus genomes that the other could not (Additional file 1: Table S1). In addition, performance of VirFinder and VirSorter were comparable for larger contigs. For such longer contigs, VirSorter may prove to be the more appropriate tool, especially if the contig contains a hallmark gene, a definitive piece of evidence for a virus. In practice, we suggest users apply both methods and analyze the overlapping and/or additive lists of probable viruses found by each tool.

As an example application of VirFinder, we assembled and identified probable viruses for human gut metagenomes of healthy and cirrhosis patients. From analysis of nearly 2700 viral contigs, viral diversity was significantly lower in diseased individuals. This is consistent with previous studies of bacterial and viral diversity of human microbiomes [3, 65, 66]. It is unclear if lower viral diversity follows lower host diversity in response to health status, vice versa, or a balanced combination of both. Longitudinal studies of patients could provide a clearer picture of which leads the other, and VirFinder would be a valuable tool for such a study. Our analysis reveals viral contigs that appear to be quite specific to particular patients, consistent with the patterns of “personalized” microbiomes observed at the prokaryotic and virus level in previous studies [7, 67, 68]. In our study, viral sequences could be used to predict with good discriminating power the health status of patients, suggesting the potential of viral microbiome analysis as a diagnostic tool. Interestingly, two of the distinguishing viral bins had sequences with similarity to crAssphage; an ubiquitous virus found in the healthy human gut microbiome. First, this suggests that there may be multiple populations of crAssphage-like viruses that have yet to be fully characterized. Second, they are both negatively associated with cirrhosis indicating that they are part of the normal status of healthy microbiomes. Furthermore, we found a putative prophage probably associated with the genus *Veillonella*, within the viral bin that was most strongly associated with liver cirrhosis patients (Table 2). This is consistent with a recent analysis of the prokaryotic component of this metagenome dataset, which found that the host genus *Veillonella* are more abundant in cirrhosis patients. This example application of VirFinder highlights the type of downstream analyses that can be done with VirFinder’s results to investigate important viral ecology questions.

## Conclusions

Our development of an innovative *k*-mer based viral identification tool adds to the increasing number of alignment-free *k*-mer based tools being utilized for analysis of large sequence datasets. In side-by-side comparison to VirSorter, VirFinder has superior performance, especially for shorter contigs (i.e., 1000 bp). Since such shorter contigs typically dominate metagenomic assemblies, VirFinder will help greatly in expanding our knowledge of natural virus communities. Our example application of VirFinder to human gut microbiomes highlights its utility in identifying diagnostic differences in viral communities between healthy and diseased individuals. Since VirFinder can be broadly applied to any type of metagenomic sample, it will be invaluable in addressing a variety of questions in the ecology of natural viral communities across various habitats types (i.e., aquatic, soil, host-associated). We also propose that VirFinder could be further implemented for identifying proviruses within large contigs, using a sliding window approach. Future integration of our *k*-mer based approach with previous gene-based tools, will further improve the accuracy and utility of virus prediction.

## Methods

### Viruses and prokaryotic host genomes used for training and validation

We collected 1562 virus RefSeq genomes infecting prokaryotes and 31,986 prokaryotic host RefSeq genomes from NCBI in May 2015. The NCBI accession numbers of the RefSeq sequences are provided in the Additional file 2: Table S2. To mimic fragmented metagenomic sequences, for a given length *L* = 500, 1000, 3000, 5000, and 10000 bp, viruses were split into non-overlapping fragments of length *L* and the same number of non-overlapping fragments of length *L* were randomly subsampled from the prokaryotic genomes. Fragments were generated for virus genomes discovered before 1 January 2014 and after 1 January 2014 and were separately used as training and testing sets, respectively (Table 1). To generate evaluation datasets containing 10, 50, and 90% viral contigs, the number of viral contigs was set as in Table 1 and was combined with 9 times more, equal numbers, or 9-fold less randomly sampled host contigs, respectively.

Highly represented host phyla (Actinobacteria, Cyanobacteria, Firmicutes, Proteobacteria) and genera (*Mycobacterium*, *Escherichia*, *Pseudomonas*, *Staphylococcus, Bacillus*, *Vibrio*, and *Streptococcus*) were selected for the analyses where viruses infecting these taxa were excluded from the training of VirFinder. For evaluation of the different trained VirFinder models, equal numbers of contigs of the excluded viruses and all other viruses were selected and then combined with randomly selected host contigs such that total virus and host contigs were equal in number.

For the analysis of VirFinder trained with 14,722 prokaryotic genomes with or without proviruses removed, these genomes were downloaded from the database cited in [6]. Likewise, the positions of proviruses predicted by VirSorter in these 14,722 genomes were obtained from the published data of [6] and were used to remove theses sequence from their corresponding host genomes.

### The *k*-mer based machine learning prediction model

For a fragment sequence *S*, let *N*(**w**) be the number of occurrences of the word **w** = *w*_1_ *w*_2_ … *w*_*k*_ and its complimentary word $\overline{\mathbf{w}}$, ${w}_i\in \mathcal{A}\equiv \left\{ A, C, G, T\right\}, i=1,2,\dots, k$. For simplicity, we use word **w** to refer to the word patterns **w** and its compliment $\overline{\mathbf{w}}$. We defined the sequence signatures as the normalized word frequencies, $V\left(\mathbf{w}\right)=\frac{N\left(\mathbf{w}\right)}{{\displaystyle {\sum}_{\boldsymbol{w}}} N\left(\mathbf{w}\right)},\mathbf{w}\in {\mathcal{A}}^k$. Duplicated word pairs were removed as in [6, 39, 69]. For example, for *k* = 4, only the 136 unique word pattern pairs are used. Based on sequence signatures, a binary classifier for identifying viral sequences was built. The classifier was trained using the training data and then was evaluated using the testing data.

Given a training dataset composed of the same number of viral sequences and host sequences, we first used *t* statistic to test for each word **w** if the mean word frequency in viral sequences was significantly different from that in host sequences. Note that *V*(***w***) were subjected to the unit sum constraint ${\displaystyle \sum_{\mathbf{w}}} V\left(\mathbf{w}\right)=1$. To overcome the problem of multicollinearity, we excluded the word with the highest *p* value (the least significant word). Then based on the selected words, we used the logistic regression model to build a binary classifier. We added a lasso regularization to make the model flexible and let the data choose the model with the highest accuracy.

Let *S*_1_, *S*_2_, …, *S*_*n*_ be *n* sequences. Let *Y*_*i*_ = 1 if *S*_*i*_ comes from viral sequences and *Y*_*i*_ = 0 if it is from host sequences, and *V*_*i*_(**w**) is the sequence signatures of *S*_*i*_, *i* = 1, …, *n*. Then we model,

$$ \log \left(\frac{P\left({Y}_i=1\Big|{V}_i\left(\mathbf{w}\right)\right)}{1- P\left({Y}_i=1\Big|{V}_i\left(\mathbf{w}\right)\right)}\right)={\displaystyle \sum_{\mathbf{w}\in {\mathcal{A}}^k}}\beta \left(\mathbf{w}\right){V}_i\left(\mathbf{w}\right) + {\beta}_0 $$,

or in other words,

$$ P\left({Y}_i=1\Big|{V}_i\left(\mathbf{w}\right)\right)=\frac{ \exp \left({\displaystyle {\sum}_{\mathbf{w}\in {\mathcal{A}}^k}}\beta \left(\mathbf{w}\right){V}_i\left(\mathbf{w}\right) + {\beta}_0\right)}{1+ \exp \left({\displaystyle {\sum}_{\mathbf{w}\in {\mathcal{A}}^k}}\beta \left(\mathbf{w}\right){V}_i\left(\mathbf{w}\right) + {\beta}_0\right)}. $$

Thus, the objective function is

$$ -\frac{1}{\mathrm{n}}{\displaystyle \sum_{i=1}^n} \log l\left({Y}_i\Big|{V}_i\left(\mathbf{w}\right),\beta \left(\mathbf{w}\right),\ {\beta}_0\right)+\lambda {\displaystyle \sum_{\boldsymbol{w}\in {\mathcal{A}}^k}}\left|\beta \left(\mathbf{w}\right)\right| $$,

where *l* is the likelihood, *β* is estimated by minimizing the objective function. We chose the parameter *λ* to have the highest AUROC using 10-fold cross validation on the training data. The R package “glmnet” was used for the model training and testing [41]. ROCs were plotted using R package “ROCR” [70] and AUC scores, and its confidence interval were computed using R package “pROC” [71].

In real metagenomic experiments, the assembled contigs are of various lengths. In order to compare scores from different prediction models, for each query contig, a *p* value was computed by comparing the score with the null distribution, that is, the distribution of scores of the testing host contigs. The *p* value was computed as the fraction of testing host contigs that have greater scores than the score of the query sequence. To estimate the false discovery rate (the proportion of predictions that are hosts), we used R package “qvalue” [37, 38] to estimate false discovery rates based on the *p* values. Then each query contig was associated with a false discovery rate, also known as the *q* value. The contigs were sorted by *q* values from the smallest to the largest. Given a threshold, the contigs with *q* values smaller than the threshold were predicted as viral sequences, and the largest *q* value among the predicted contigs gave the estimation of the false discovery rate for the prediction.

### Simulation studies on metagenomes

Metagenomic samples were simulated based on species abundance profiles derived from a real human gut metagenomic sample (accession ID SRR061166, Platform: Illumina) from the Human Microbiome Project (HMP) [72], commonly used for metagenomic data analysis [73–76].

Following a similar simulation procedure as in [77], we first mapped reads from sample SRR061166 using bwa-0.7.15 [78] to 1562 virus and 2698 host complete genome sequences downloaded from NCBI RefSeq to generate abundance profiles. The reads from the sample were first mapped to viral genomes and then the remaining unmapped reads were mapped to the host complete genomes using the command of “bwa mem”. 10% of reads mapped to viral genomes, consistent with the range previously reported for human gut metagenomes (4–17% viral) [7]. The abundance profiles are provided in the Additional file 3: Table S3. Then we used NeSSM [33] to simulate metagenomic samples with pair-end short reads of length 150 bp in an Illumina MiSeq setting mode based on the abundance profiles. Ten and 20 million read samples were generated at 3 different mixtures of virus and prokaryotic sequences. The relative abundance among viruses and among hosts were kept the same and the virus and hosts reads were mixed to make 10 (the native level in sample SRR061166 determined from mapping), 50, and 90% viral samples. metaSPAdes [34, 35] was used to de-novo assemble the simulated metagenome samples, using the command “spades.py –meta”. Only contigs ≥500 bp were used for the downstream analysis.

To obtain the true labels of the assembled contigs, reads in the simulated data were mapped to the set of contigs using “bwa mem”. A contig was labeled as a viral contig if it was assembled from reads only from viral genomes; similarly, a contig was labeled as a host contig if it was assembled from reads from host genomes. A contig was labeled as chimeric if it was assembled from a mixture of virus and host reads. To validate the prediction, we plotted ROC (receiver operating characteristic) curves at different ranges of contig lengths, 500–1000 bp, 1000–3000 bp and ≥3000 bp. The ROC curves were based on the predictions of viral contigs from genomes sequenced after 1 January 2014 paired with the same number of randomly sampled host contigs.

### VirSorter settings

VirSorter was run in the “Viromes” mode on the same sets of evaluation sequences as used for VirFinder. VirSorter reported predicted viral sequences in three categories: I for “most confident” predictions, II for “likely” predictions, and III for “possible” predictions.

### Assembly and analysis of human gut metagenomic samples from liver cirrhosis study

The data from Qin et al. [36] contains two independent datasets each of which contains Illumina 2 × 100 bp paired read metagenomes of stool samples from both healthy individuals and liver cirrhosis patients, all of Han Chinese origin. These metagenomes were downloaded from the European Nucleotide Archive, accession number ERP005860. The first dataset, referred to as the “training set”, has 78 samples comprised of 40 samples from 31 healthy patients and 38 samples from 25 liver cirrhosis patients. The second dataset, referring to as the “testing set”, has 230 samples comprised of 103 samples from 83 healthy patients and 127 samples from 98 liver cirrhosis patients.

Megahit [79] was used to cross-assemble the 78 sample training dataset using the default settings since the 230 sample dataset was too large for assembly. COCACOLA [39] was used to separately cluster viral contigs predicted by VirFinder and VirSorter based on sequence tetranucleotide frequencies and contig coverages normalized by contig length and number of mapped reads in samples. Contig coverages (RPKMs) were determined by mapping sample reads with Bowtie2 [80] using the default settings and were averaged for each bin. Averaged bin RPKMs were used to train a classification model to classify the disease status (0 for healthy and 1 for liver cirrhosis). A logistic regression model with lasso regularization was used in order to enhance the prediction accuracy and interpretability. Thus, a subset of viral bins was chosen to achieve the best prediction accuracy. To assess the classification model, the average RPKM of bins in the second dataset with 230 samples were used to test the classification model, and ROC curves were used for evaluation. Two-way hierarchical clustering was performed using the average RPKM coverages of the 116 VirFinder contig bins using all 78 training set samples and 78 samples randomly selected from the 230 sample testing dataset. Distances were computed using Euclidean distance and were clustered with complete linkage method in R.

Blast analyses were used to assess if predicted viral contigs assembled from the cirrhosis study samples had similarity to previously reported sequences. Blastn and blastp searches were performed with default settings against NCBI’s non-redundant nucleotide (nt) and protein (nr) databases from August 2016. Protein sequences were predicted for each contig using Prodigal [81, 82] with the “meta” procedure (−p meta). The best hits for each contig (nucleotide) or each predicted protein on the contigs were retained. Resulting proteins in the nr databases were called as viral if they came from a virus (recorded in their taxonomy) or had one of the following terms in their definition lines: virus, phage, capsid, tail, head, or terminase. Proteins were also searched against Pfam domains via the webserver at http://pfam.xfam.org/. Resulting domains were considered viral if their description contained one of the following terms: virus, phage, capsid, tail, head, tape, terminase, Gp*nn* (where *n* are digits), “podo”, or “sipho”.

## Additional files

Additional file 1: Supplemental material for Ren et al. "VirFinder: a novel k-mer based tool for identifying viral sequenvces from assembled metagenomic data". (PDF 3007 kb)

Additional file 2: Table S2. NCBI accession numbers for prokaryotic host and viral genomes used in the training and evaluation of VirFinder. (XLSX 2752 kb)

Additional file 3: Table S3. The virus and prokaryotic species abundance profile used for the simulation of metagenomic samples.(XLSX 164 kb)

Additional file 4:Table S4. Information about the 2,657 top-scoring predicted viral contigs assembled from 78 human gut microbiome samples from the liver cirrhosis study of Qin et al. 2014. (XLSX 520 kb)
