# Supplementary material for: VirFinder: a novel k-mer based tool for identifying viral sequences from assembled metagenomic data
Source: Microbiome. 2017 Jul 6;5:69. doi: 10.1186/s40168-017-0283-5 (PMC5501583; doi:10.1186/s40168-017-0283-5)
Supplement: Supplementary file 1 — Supplemental material for Ren et al. "VirFinder: a novel k-mer based tool for identifying viral sequenvces from assembled metagenomic data". (PDF 3007 kb) [file 40168_2017_283_MOESM1_ESM.pdf]

**Supplemental Material for Ren et al. "VirFinder: a novel *k*-mer based tool for identifying viral sequences from assembled metagenomic data"**

**Table S1.** VirFinder and VirSorter prediction results for $n$=45 RefSeq prokaryotic virus genomes sequenced after 1/1/2014 that have no significant blastn similarity (E-value > $1e^{-5}$) to RefSeq prokaryotic virus genomes sequenced before 1/1/2014.

| Virus genome | NCBI accession | Length (bp) | VirFinder score | VirFinder *p*-value | VirSorter result[a] | Evaluate VirFinder (VF) and VirSorter (VS) results[b] |
|---|---|---|---|---|---|---|
| *Oenococcus* phage phi9805 | NC_023559_1 | 46145 | 0.060 | 0.422 | II | VS but not VF |
| *Oenococcus* phage phiS11 | NC_023571_1 | 46243 | 0.065 | 0.407 | II | VS but not VF |
| *Oenococcus* phage phiS13 | NC_023560_1 | 43454 | 0.094 | 0.345 | II | VS but not VF |
| Eel River basin pequenovirus isolate c22476 | NC_026665_1 | 6083 | 0.255 | 0.187 | II | VS but not VF |
| *Mycobacterium* phage Adler | NC_023591_1 | 95705 | 0.401 | 0.120 | Cat. VI provirus | Neither |
| *Vibrio* phage X29 | NC_024369_2 | 41569 | 0.565 | 0.073 | II | VS but not VF |
| Uncultured phage WW-nAnB strain 3 | NC_026613_1 | 5210 | 0.657 | 0.054 | N | Neither |
| *Shewanella sp.* phage 1/41 | NC_025458_1 | 43510 | 0.686 | 0.049 | II | VS but not VF |
| *Rhizobium* phage vB_RglS_P106B | NC_023566_1 | 56024 | 0.696 | 0.047 | II | VS but not VF |
| *Psychrobacter* phage Psymv2 | NC_023734_1 | 35725 | 0.704 | 0.045 | II | VS but not VF |
| *Erwinia* phage Ea35-70 | NC_023557_1 | 271084 | 0.765 | 0.034 | II | VS but not VF |
| *Arthrobacter* phage vB_ArtM-ArV1 | NC_026606_1 | 71200 | 0.803 | 0.028 | II | VS but not VF |
| *Vibrio* phage VpKK5 | NC_026610_2 | 56637 | 0.894 | 0.014 | II | VS but not VF |
| Microviridae IME-16 | NC_026013_1 | 5755 | 0.917 | 0.012 | III | Neither |
| *Croceibacter* phage P2559Y | NC_023614_1 | 43153 | 0.924 | 0.010 | II | VS but not VF |
| *Lactococcus* phage WP-2 | NC_024149_1 | 18899 | 0.934 | 0.009 | II | Both |
| *Aeromonas* phage pAh6-C | NC_025459_1 | 53744 | 0.944 | 0.008 | II | Both |
| *Rhizobium* phage vB_RleS_L338C | NC_023502_1 | 109558 | 0.946 | 0.008 | II | Both |
| *Rhodococcus* phage ReqiPoco6 | NC_023694_1 | 78064 | 0.948 | 0.007 | II | Both |
| *Erwinia* phage PhiEaH1 | NC_023610_1 | 218339 | 0.951 | 0.007 | II | Both |
| Uncultured phage WW-nAnB strain 2 | NC_026612_1 | 5077 | 0.954 | 0.007 | N | **VF but not VS** |
| *Rhodococcus* phage ReqiPepy6 | NC_023735_1 | 76797 | 0.957 | 0.007 | II | Both |
| *Idiomarinaceae* phage Phi1M2-2 | NC_025471_1 | 36844 | 0.957 | 0.007 | II | Both |
| *Shewanella sp.* phage 3/49 | NC_025466_1 | 40161 | 0.963 | 0.006 | II | Both |
| *Idiomarinaceae* phage 1N2-2 | NC_025439_1 | 34773 | 0.966 | 0.005 | II | Both |

| | | | | | | |
|---|---|---|---|---|---|---|
| *Clavibacter* phage CN1A | NC_023549_1 | 56789 | 0.969 | 0.005 | II | Both |
| Uncultured phage WW-nAnB | NC_026582_1 | 4817 | 0.972 | 0.005 | N | **VF but not VS** |
| *Ruegeria* phage DSS3-P1 | NC_025428_1 | 59601 | 0.973 | 0.005 | II | Both |
| *Vibrio* phage CHOED | NC_023863_2 | 66316 | 0.975 | 0.004 | II | Both |
| *Shewanella sp.* phage 1/44 | NC_025463_1 | 49640 | 0.975 | 0.004 | II | Both |
| *Mesorhizobium* phagevB_MloP_Lo5R7ANS | NC_025431_1 | 45718 | 0.976 | 0.004 | II | Both |
| *Shewanella* phage Spp001 | NC_023594_1 | 54789 | 0.979 | 0.004 | II | Both |
| *Enterococcus* phage vB_Efae230P-4 | NC_025467_1 | 17972 | 0.980 | 0.004 | II | Both |
| Uncultured phage crAssphage | NC_024711_1 | 97065 | 0.983 | 0.003 | II | Both |
| *Rhizobium* phage vB_RleM_P10VF | NC_025429_1 | 156446 | 0.986 | 0.003 | II | Both |
| *Vibrio* phage SHOU24 | NC_023569_1 | 77837 | 0.988 | 0.003 | III | **VF but not VS** |
| *Acinetobacter* phage IME_AB3 | NC_023590_1 | 43050 | 0.990 | 0.002 | II | Both |
| *Rhodococcus* phage ReqiDocB7 | NC_023706_1 | 75772 | 0.992 | 0.002 | II | Both |
| *Microbacterium* phage vB_MoxS-ISF9 | NC_023859_1 | 59254 | 0.991 | 0.002 | II | Both |
| *Shewanella sp.* phage 1/40 | NC_025470_1 | 139004 | 0.991 | 0.002 | II | Both |
| *Shewanella sp.* phage 1/4 | NC_025436_1 | 133824 | 0.993 | 0.002 | II | Both |
| *Acinetobacter* phage vB_AbaM_Acibel004 | NC_025462_1 | 99730 | 0.996 | 0.001 | Cat. V provirus | Both |
| *Pseudomonas* phage phi_Pto-bp6g | NC_023718_1 | 26499 | 0.997 | 0.001 | III | **VF but not VS** |
| *Vibrio* phage phi-A318 | NC_025822_1 | 42544 | 0.999 | 0.000 | II | Both |
| *Anabaena* phage A-4L | NC_024358_1 | 41750 | 1.000 | 0.000 | II | Both |

a – VirSorter prediction result (category I, II, or III, for viruses and IV, V, and VI for category 1, 2, 3 results for detected proviruses). N = no prediction made by VirSorter.
b – Summary of the results comparing the VirFinder (VF, *p*-value < 0.01) and VirSorter (VS, only cat. I & II predictions) results. "Neither" = neither method predicted the genome as viral, "Both" = both VF and VS predicted it as viral, "VF but not VS" = VF predicted it as viral but VS did not, and "VS but not VF" = VS predicted it as viral but VF did not

**Table S2.** NCBI accession numbers for prokaryotic host and viral genomes used in the training and evaluation of VirFinder. This table is available as supplemental online material.

**Table S3.** Table of abundances of 1,562 possible virus and 2,698 complete prokaryotic genomes as determined by read mapping of human gut microbiome metagenome sample SRR061166 from Vázquez-Castellanos et al. 2014. These abundances were used to generate simulated metagenomes. This table is available as supplemental online material.

**Table S4.** Information about the 2,657 top-scoring predicted viral contigs assembled from 78 human gut microbiome samples from the liver cirrhosis study of Qin et al. 2014.

The table includes general information about the size of contigs and to which bin they belong, VirFinder and VirSorter prediction results, and whether or not those contigs have significantly similarity to other sequences in NCBI's nucleotide nt and protein nr databases. This table is available as supplemental online material.

## Supplemental Figure Legends

**Figure S1.** Area under the receiver operator curve (AUROC) (A) and Area under precision-recall curves (AUPRC) (B) for predictions results made with VirFinder on varying mixtures of viral and host contigs subsampled from viral and host genomes sequenced after 1/1/2014. VirFinder was trained using contigs equal numbers of viral and host contigs subsampled from genomes sequenced before 1/1/2014 as in the results for Fig. 1. Bars depict mean values for 30 replicate bootstrap samples and error bars depict the standard error.

**Figure S2**. Performance of VirSorter and VirFinder virus prediction for contigs subsampled from virus and prokaryotic genomes as in Fig. 2A, except that evaluation datasets contained 10% (A) or 90% (B) viral contigs. Results are shown for the fraction of true viral contigs (true positive rate, TPR) when using VirSorter category I and II predictions and VirFinder at the same false positive rate (FPR) as VirSorter (listed in or above the VirSorter bars) and at FPRs of 0.001, 0.005, and 0.01. Bars depict mean values for 30 replicate bootstrap samples and error bars depict the standard error. TPRs of VirFinder were all significantly higher than that of VirSorter at the same false positive rate (Wilcoxon signed-rank one sided test, $p < 0.001$).

**Figure S3.** Sensitivity of VirFinder to random mutations applied to evaluation contigs. VirFinder prediction results as evaluated by AUROCs were determined on contigs subsampled from viral and host genomes with no mutations applied vs. when random mutations were applied to the contigs at three different rates (0.0001, 0.001, and 0.01 substitutions per position). Bars represent averages of 30 replicate datasets tested, and error bars indicate standard deviations. Within each contig length group, there was only a significant difference in values between the 0.01 rate and the case of no mutation ($p < 0.01$, *t*-test).

**Figure S4.** Precision-recall curves and AUPRC for VirFinder results when analyzing contigs assembled from simulated metagenomes. (A) Precision-recall curves for the analysis of equal proportions of viral and host contigs representing genomes sequenced after 1/1/2014. Results are shown for when chimeras were included or excluded from the analysis. (B) AUPRC scores for various VirFinder results when varying the total sequencing depth for the simulated metagenomes (10 M or 20 M reads) and varying the relative abundance of viral and host contigs analyzed. Bars represent averages of 30 replicate datasets tested, and error bars indicate standard deviations.

**Figure S5**. Evaluation of VirFinder (VF) and VirSorter (VS) predictions on contigs for three length ranges assembled from simulated human gut metagenomes when viral contigs were combined with host contigs at 10% (A) and 90% (B) viral levels. Bars depict true positive rates (TPRs) for VirSorter category I; I and II; and I, II, and III predictions. As in Fig. 5, VirFinder predictions were evaluated at the same false positive rates (FPRs) as corresponding VirSorter results. Thirty replicate bootstrap samples of contigs assembled from simulated metagenomes were tested for each condition. Metagenomes were simulated based on the relative abundance of complete virus and host genomes found in a real human gut metagenome. The horizontal bar displays the median, boxes display the first and third quartiles, and whiskers depict minimum and maximum values. "*" indicates VirFinder's TPRs are significantly larger than VirSorter's (Wilcoxon signed-rank one sided test, $p < 10^{-5}$).

**Figure S6.** Evaluation of VirFinder (VF) and VirSorter (VS) predictions on contigs assembled from simulated human gut metagenomes when viral contigs were combined with host contigs at 10%, 50% and 90% viral levels. Results are shown for predictions made on all contigs > 500 bp (left column) or all contigs > 1000 bp (right column). Bars depict true positive rates (TPRs) for VirSorter category I ("I"); I and II ("I&II"); and I, II, and III ("I-III") predictions. As in Fig. 5, VirFinder predictions were evaluated at the same false positive rates (FPR) as corresponding VirSorter results. Thirty replicate bootstrap samples of contigs assembled from simulated metagenomes were tested for each condition. Metagenomes were simulated based on the relative abundance of complete virus and host genomes found in a real human gut metagenome. The horizontal bar displays the median, boxes display the first and third quartiles, and whiskers depict minimum and maximum values. "*" indicates VirFinder's TPRs are significantly larger than VirSorter's (Wilcoxon signed-rank one sided test, $p < 0.05$).

**Figure S7.** Histogram of the lengths of 352,020 contigs that are >1,000 bp generated by cross-assembly of 78 human gut metagenomic samples from 40 healthy and 38 liver cirrhosis patients (Qin et al. 2014).

**Figure S8.** Histograms depicting the cumulative frequencies for different groups of *k*-mers (length 8) as they occur in viral and host contigs. Panels depict the top 100, 500, 1000 most highly scored *k*-mers or all *k*-mers used by VirFinder (trained with 1,000 bp contigs) to generate prediction scores ($n$=6269 and 6082 for *k*-mers with positive and negative coefficients respectively). The left column of graphs depicts *k*-mers with positive coefficients in VirFinder's model (i.e. those that are found more frequently among viral sequences) and the right column shows *k*-mers that are negatively scored (those that are found more frequently among host sequences). In each panel, host and viral *k*-mer distributions were significantly different ($p < 10^{-16}$, *t*-test).

**Figure S9.** Similarity between the prediction proteins on the crAssphage genome (below) and on two contigs belonging to viral bin 64 (above). Grey arrows depict predicted proteins and trapezoids depict the percent amino acid identity between two connected

genes as determined by blastp searches. Numbers in crAssphage genes indicate the annotated locus tag of those genes (UGP_xxx).

**Figure S10.** VirFinder predictions were made when it was trained on the set of 14,722 prokaryotic host genomes from Roux et al. 2015 and the 1,225 viral genomes sequenced before 1/1/2014 that were used in the rest of our study. The Roux et al. host genomes were used as is or with proviruses identified by VirSorter removed (‘proviruses removed’). VirFinder predictions were made on contigs with various lengths of virus genomes sequenced after 1/1/2014 and host genomes subsampled from host genomes after 1/1/2014 at equal proportions, and the resulting AUROC values are shown. The difference in AUROC values among the three datasets are less than 3%. Bars depict the mean of results on 30 replicate evaluation datasets and error bars depict standard deviations.

**Figure S11.** VirFinder predictions were made when VirFinder was trained with viral and prokaryotic sequences as before or with viral contigs ‘spiked’ into the host training set to assess the impact of an overabundance of proviruses in host training dataset. VirFinder was trained on host and viral contigs that were subsampled at equal numbers from prokaryotic and viral genomes sequenced before 1/1/2014 (“Control”) and when 5% of the host contigs in the training set were replaced with contigs subsampled from viral genomes (“5% viral contigs added to host training database”). Predictions were made on equal numbers of viral and host contigs subsampled from genomes sequenced after 1/1/2014. Bar depict mean AUROC values for 30 replicate sets of subsampled contigs and error bars depict standard deviations.

# Figure S1

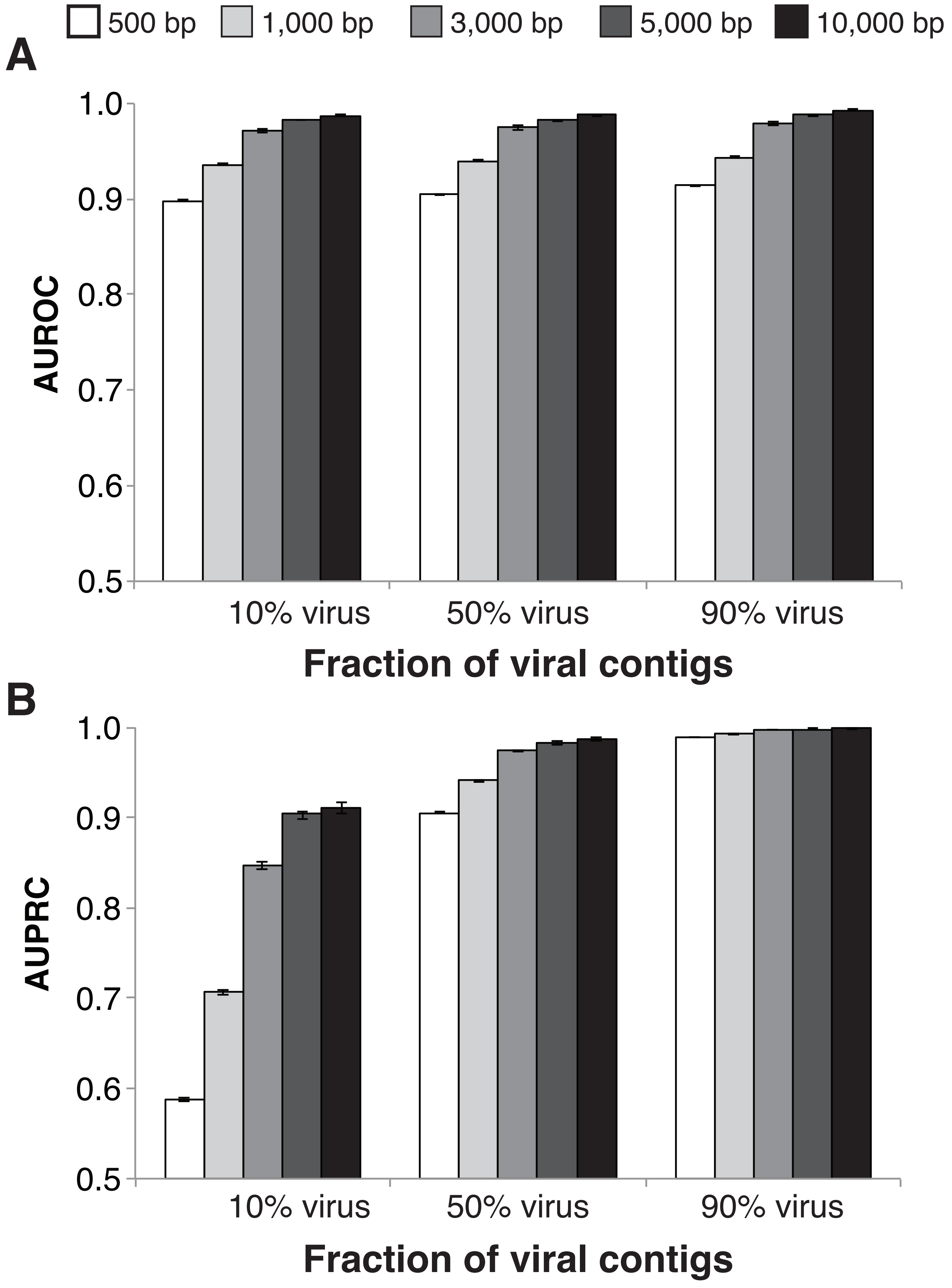
500 bp
1,000 bp
3,000 bp
5,000 bp
10,000 bp
A
AUROC
1.0
0.9
0.8
0.7
0.6
0.5
10% virus
50% virus
90% virus
Fraction of viral contigs
B
AUPRC
1.0
0.9
0.8
0.7
0.6
0.5
10% virus
50% virus
90% virus
Fraction of viral contigs

## Figure S2

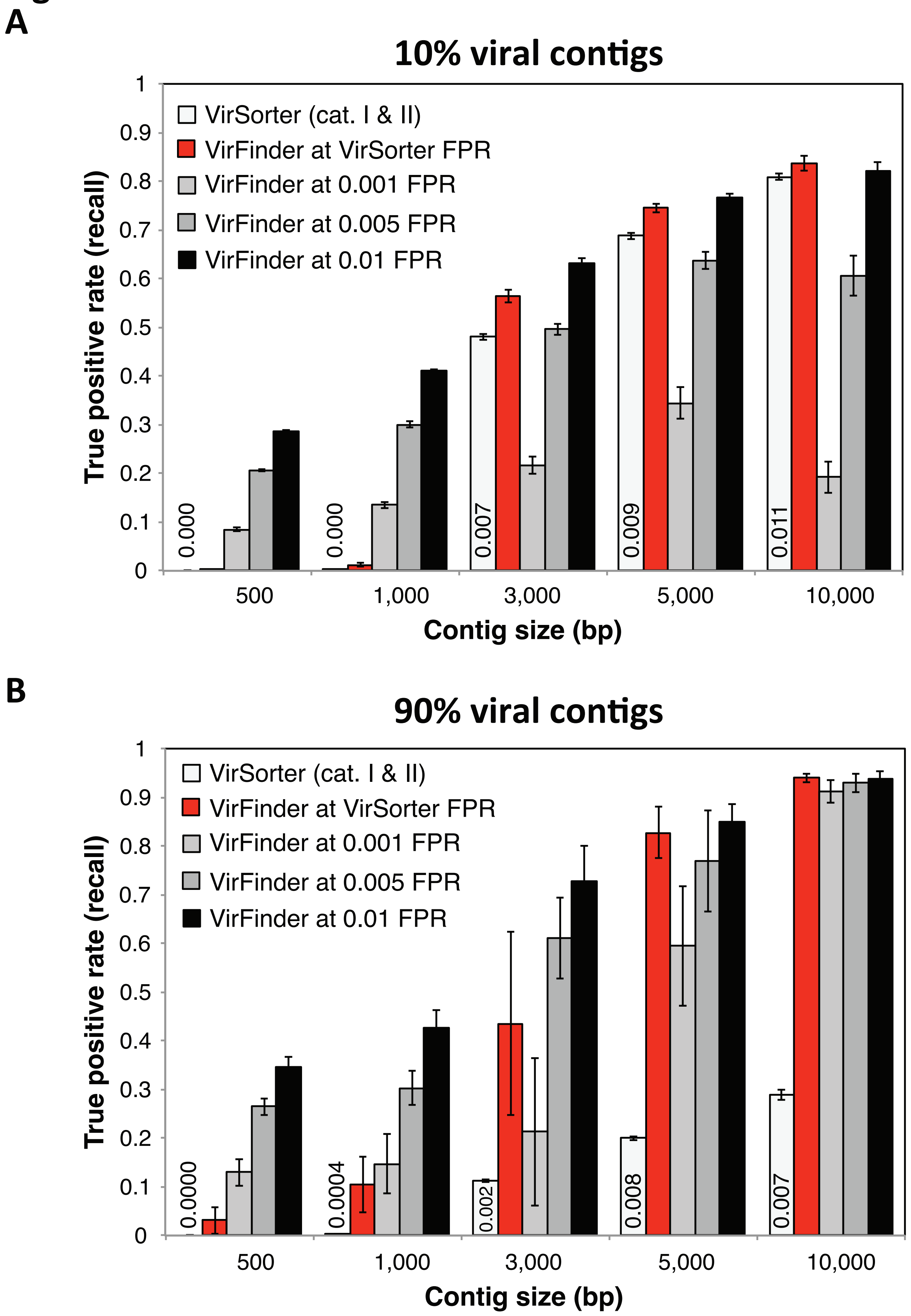
A
10% viral contigs
True positive rate (recall)
0
0.1
0.2
0.3
0.4
0.5
0.6
0.7
0.8
0.9
1
VirSorter (cat. I & II)
VirFinder at VirSorter FPR
VirFinder at 0.001 FPR
VirFinder at 0.005 FPR
VirFinder at 0.01 FPR
0.000
0.000
0.007
0.009
0.011
500
1,000
3,000
5,000
10,000
Contig size (bp)
B
90% viral contigs
True positive rate (recall)
0
0.1
0.2
0.3
0.4
0.5
0.6
0.7
0.8
0.9
1
VirSorter (cat. I & II)
VirFinder at VirSorter FPR
VirFinder at 0.001 FPR
VirFinder at 0.005 FPR
VirFinder at 0.01 FPR
0.0000
0.0004
0.002
0.008
0.007
500
1,000
3,000
5,000
10,000
Contig size (bp)

# Figure S3

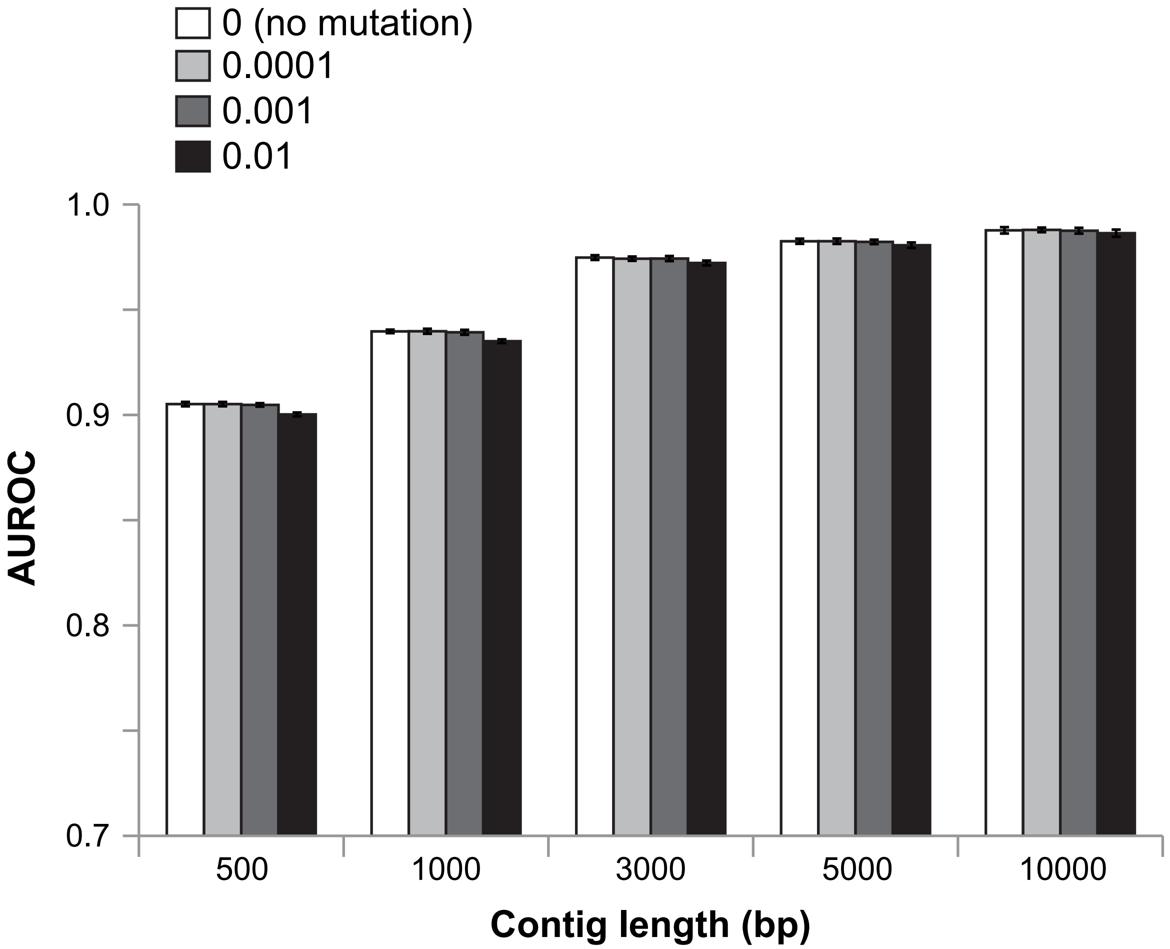
0 (no mutation)
0.0001
0.001
0.01
1.0
0.9
0.8
0.7
AUROC
500
1000
3000
5000
10000
Contig length (bp)

# Figure S4

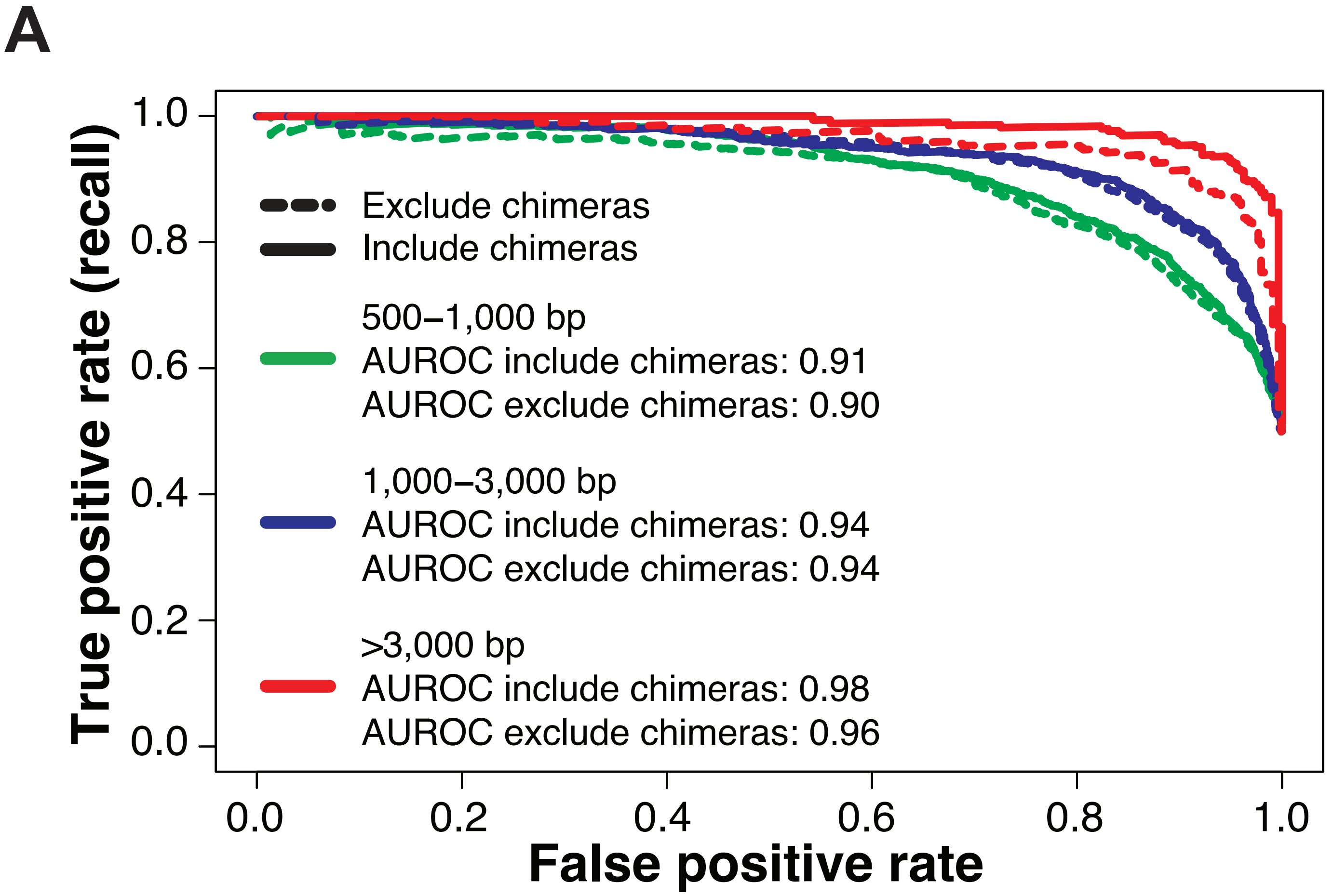

A
True positive rate (recall)
1.0
0.8
0.6
0.4
0.2
0.0
Exclude chimeras
Include chimeras
500–1,000 bp
AUROC include chimeras: 0.91
AUROC exclude chimeras: 0.90
1,000–3,000 bp
AUROC include chimeras: 0.94
AUROC exclude chimeras: 0.94
>3,000 bp
AUROC include chimeras: 0.98
AUROC exclude chimeras: 0.96
0.0
0.2
0.4
0.6
0.8
1.0
False positive rate


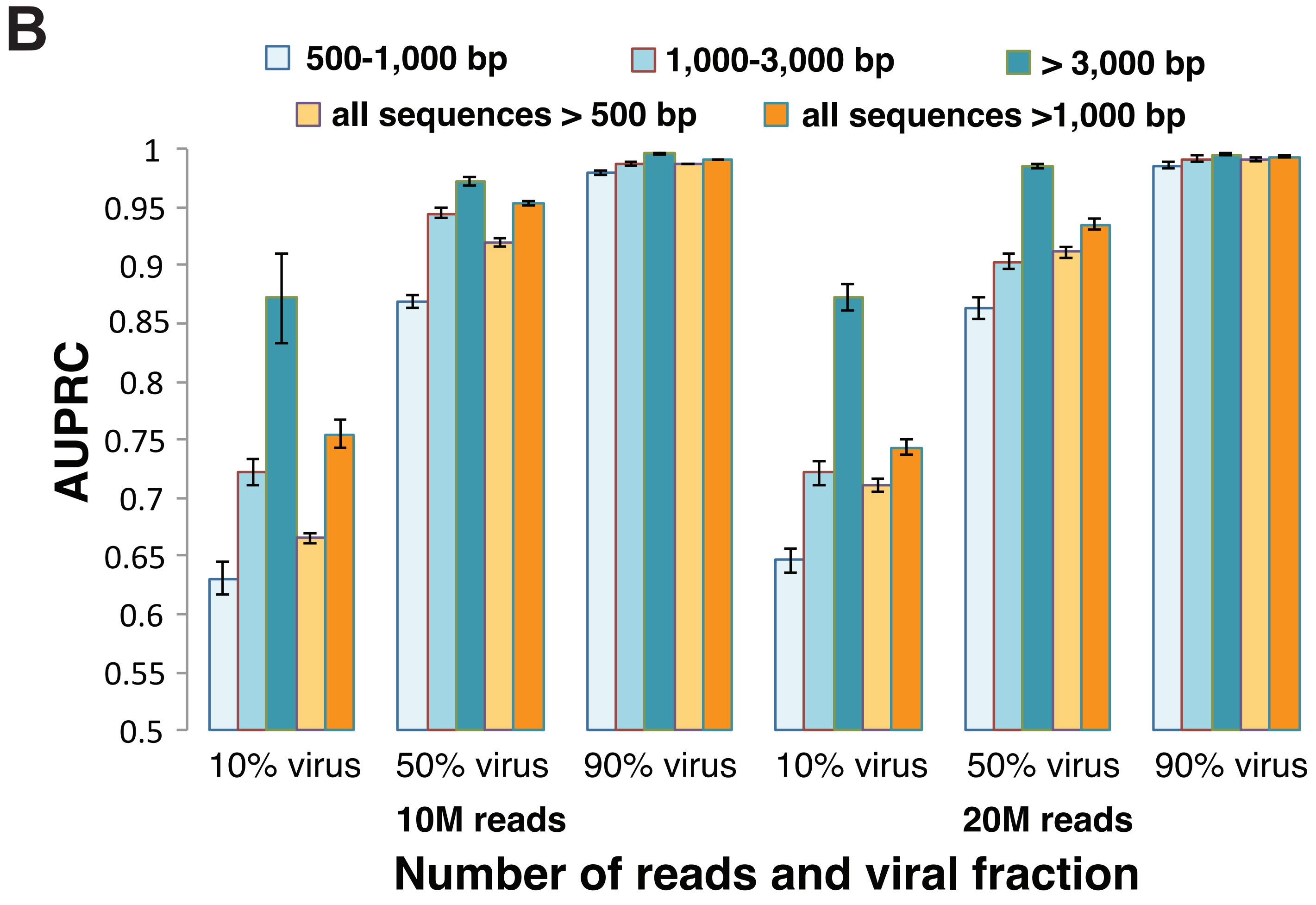

B
500-1,000 bp
1,000-3,000 bp
> 3,000 bp
all sequences > 500 bp
all sequences >1,000 bp
AUPRC
1
0.95
0.9
0.85
0.8
0.75
0.7
0.65
0.6
0.55
0.5
10% virus
50% virus
90% virus
10% virus
50% virus
90% virus
10M reads
20M reads
Number of reads and viral fraction

# Figure S5

## A 10% viral contigs

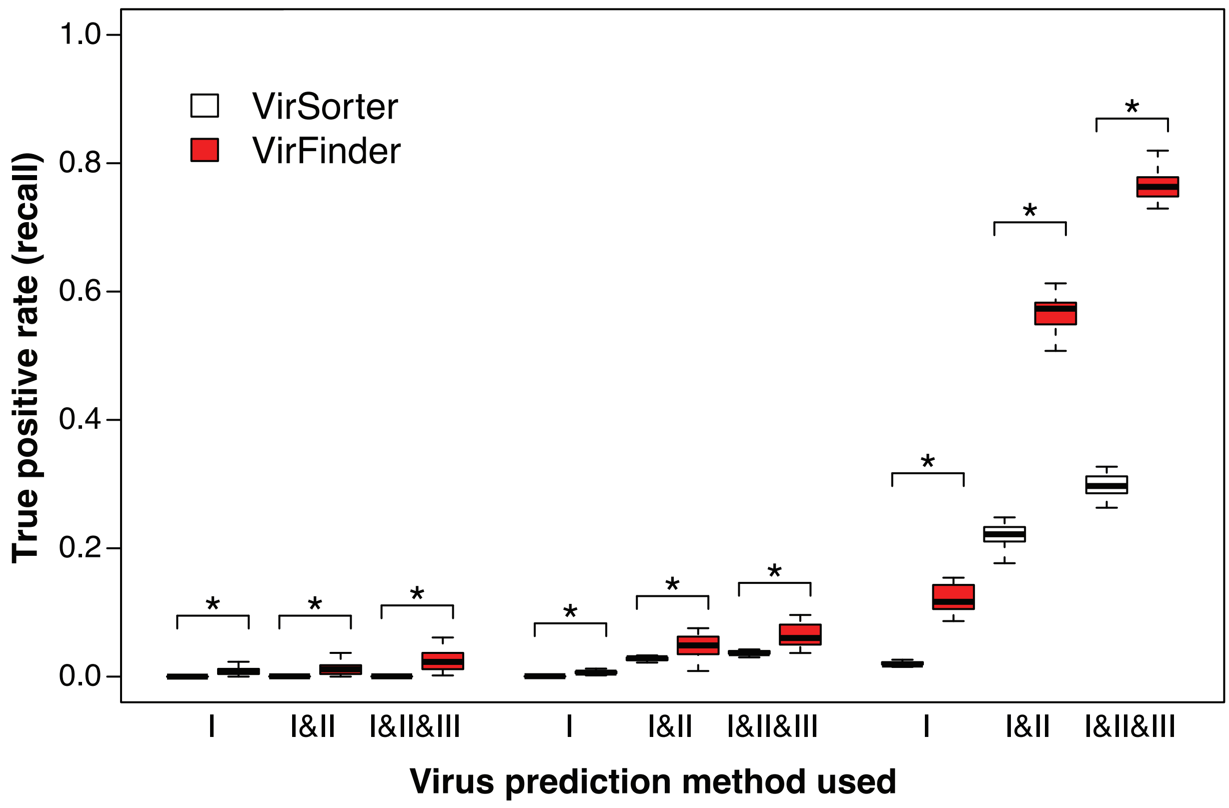


## B 90% viral contigs

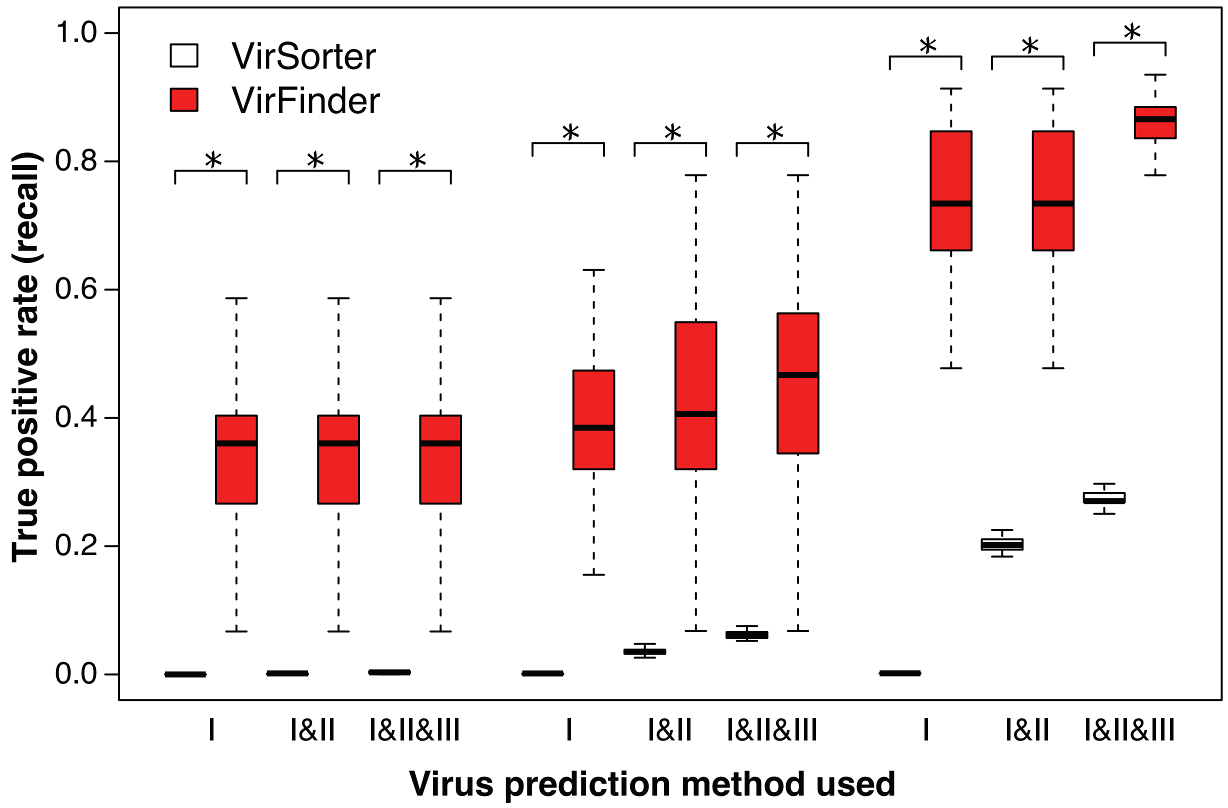

# Figure S6

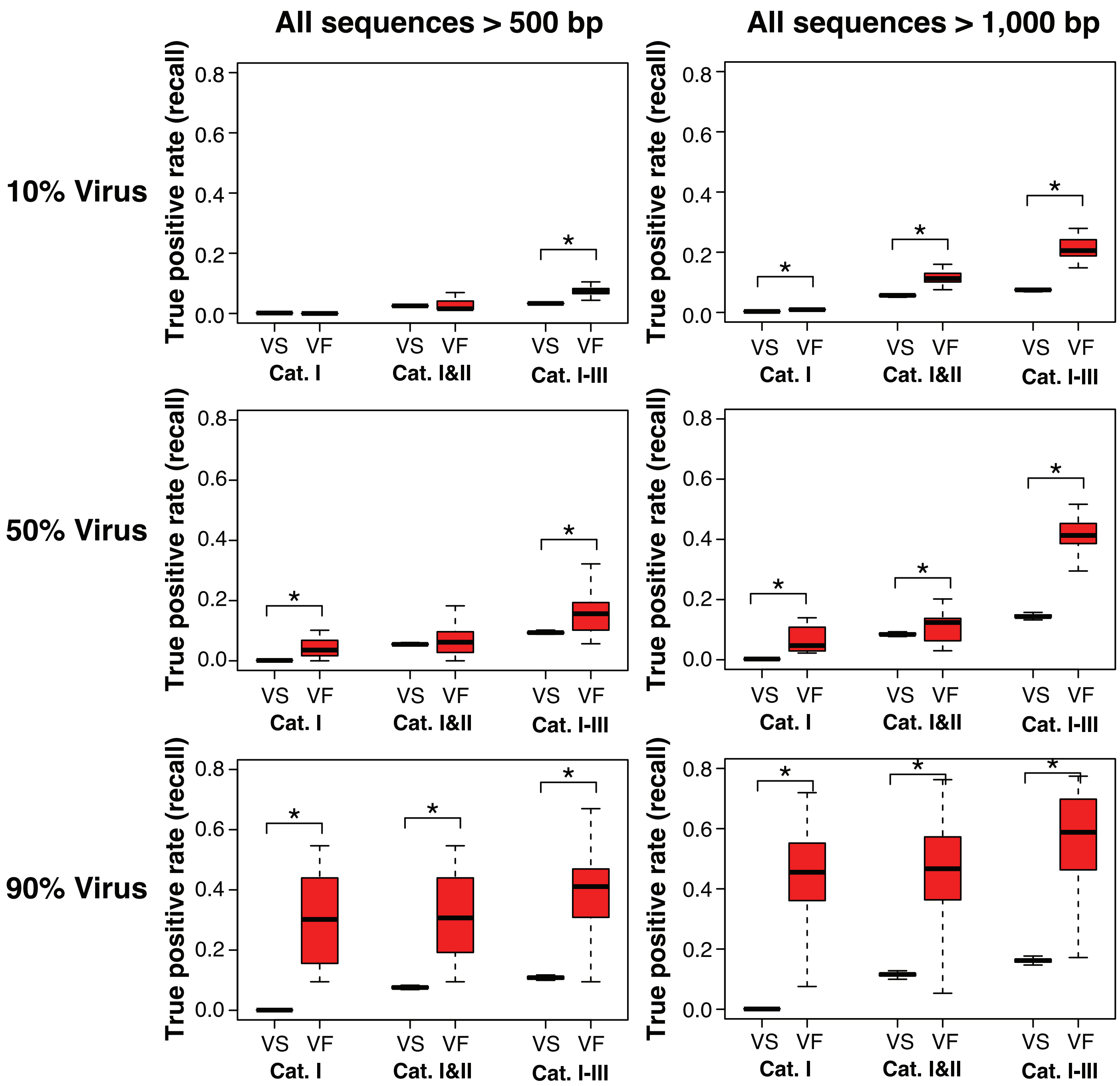
All sequences > 500 bp
All sequences > 1,000 bp
10% Virus
50% Virus
90% Virus
True positive rate (recall)
0.0
0.2
0.4
0.6
0.8
VS
VF
Cat. I
Cat. I&II
Cat. I-III
*

# Figure S7

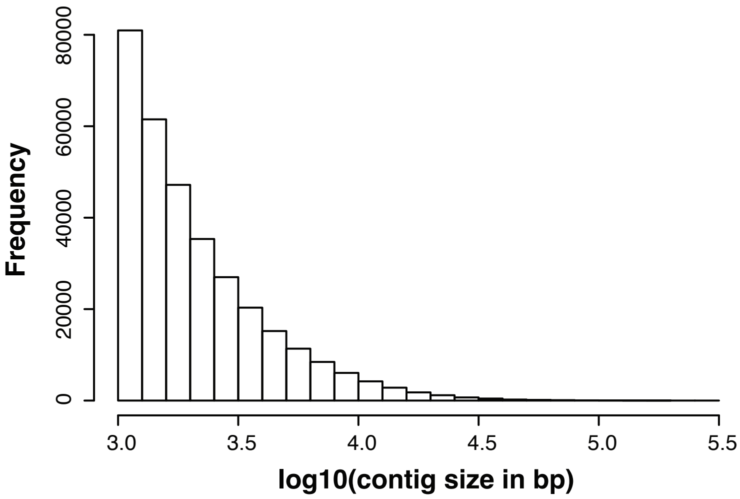

Frequency
80000
60000
40000
20000
0
3.0
3.5
4.0
4.5
5.0
5.5
log10(contig size in bp)

# Figure S8

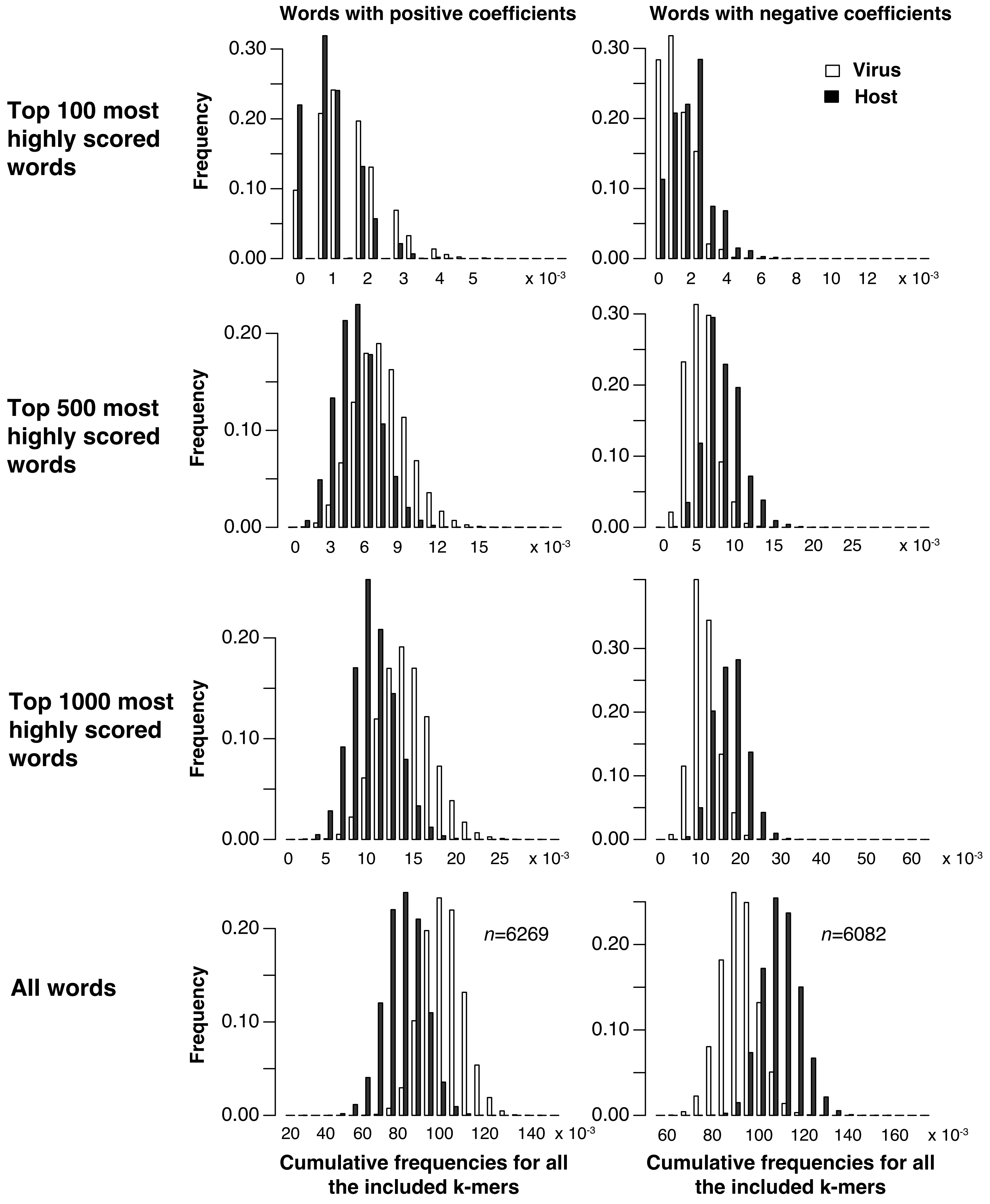

Words with positive coefficients
Words with negative coefficients
Top 100 most highly scored words
Top 500 most highly scored words
Top 1000 most highly scored words
All words
Frequency
Virus
Host
x 10-3
n=6269
n=6082
Cumulative frequencies for all the included k-mers

# Figure S9

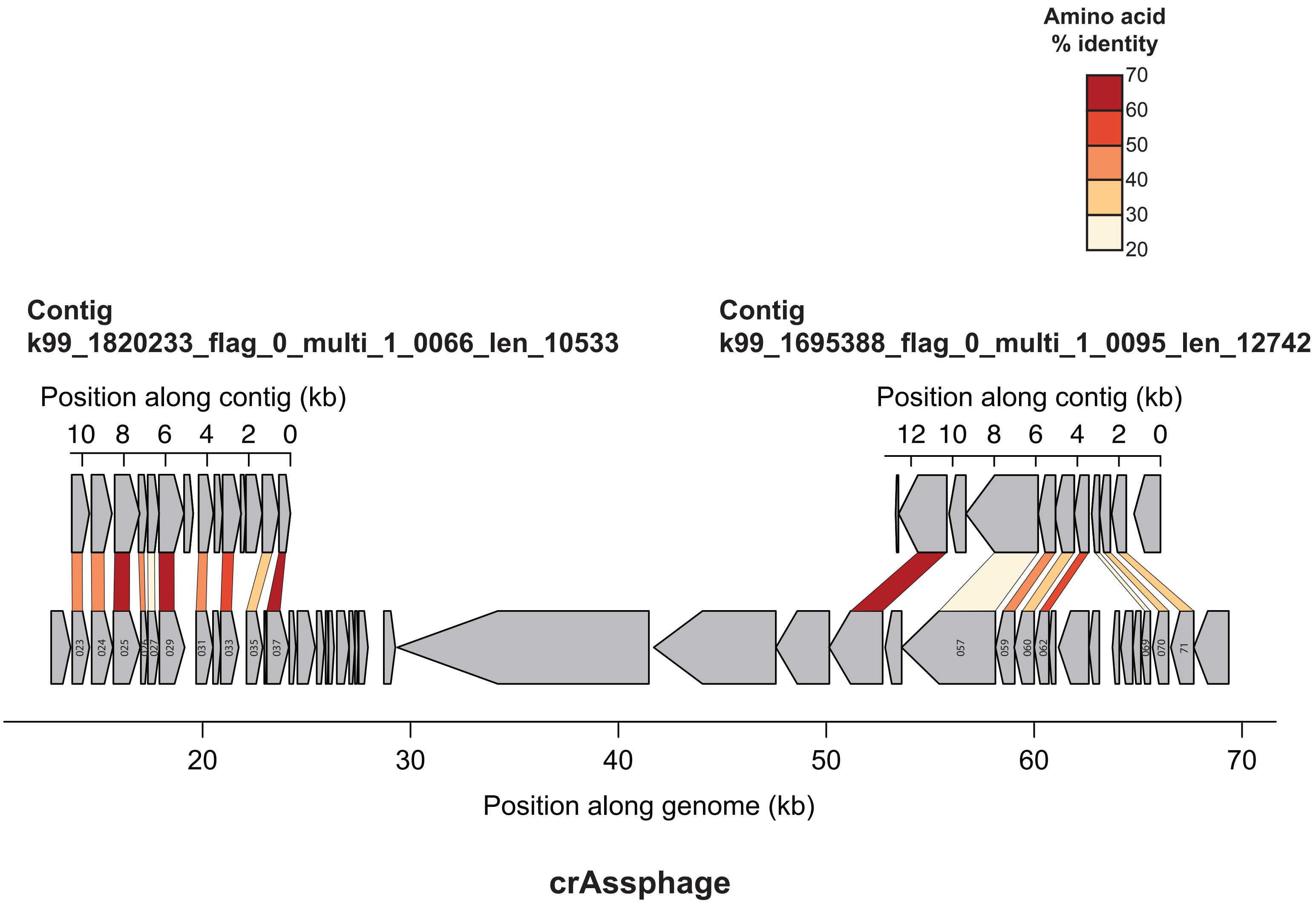

Amino acid % identity
70
60
50
40
30
20
Contig k99_1820233_flag_0_multi_1_0066_len_10533
Contig k99_1695388_flag_0_multi_1_0095_len_12742
Position along contig (kb)
10 8 6 4 2 0
Position along contig (kb)
12 10 8 6 4 2 0
20
30
40
50
60
70
Position along genome (kb)
crAssphage

## Figure S10

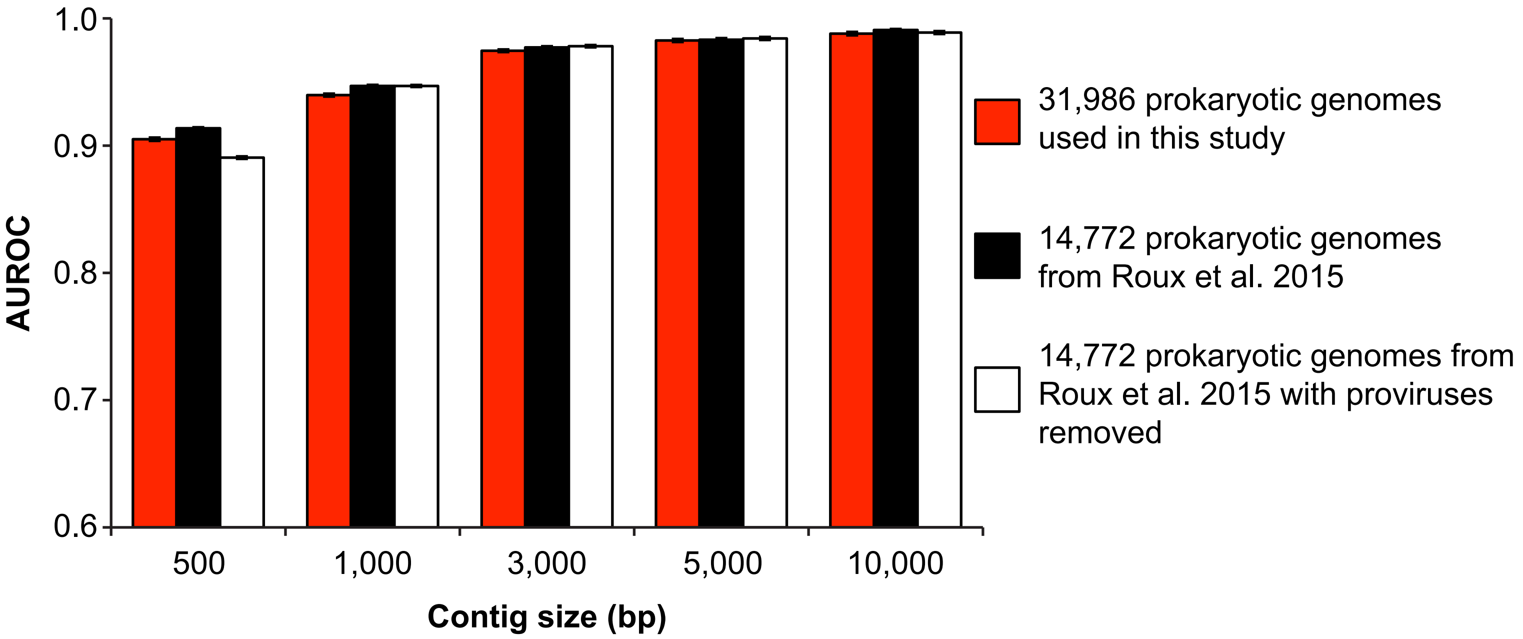

1.0
0.9
0.8
0.7
0.6
AUROC
500
1,000
3,000
5,000
10,000
Contig size (bp)
31,986 prokaryotic genomes used in this study
14,772 prokaryotic genomes from Roux et al. 2015
14,772 prokaryotic genomes from Roux et al. 2015 with proviruses removed

# Figure S11

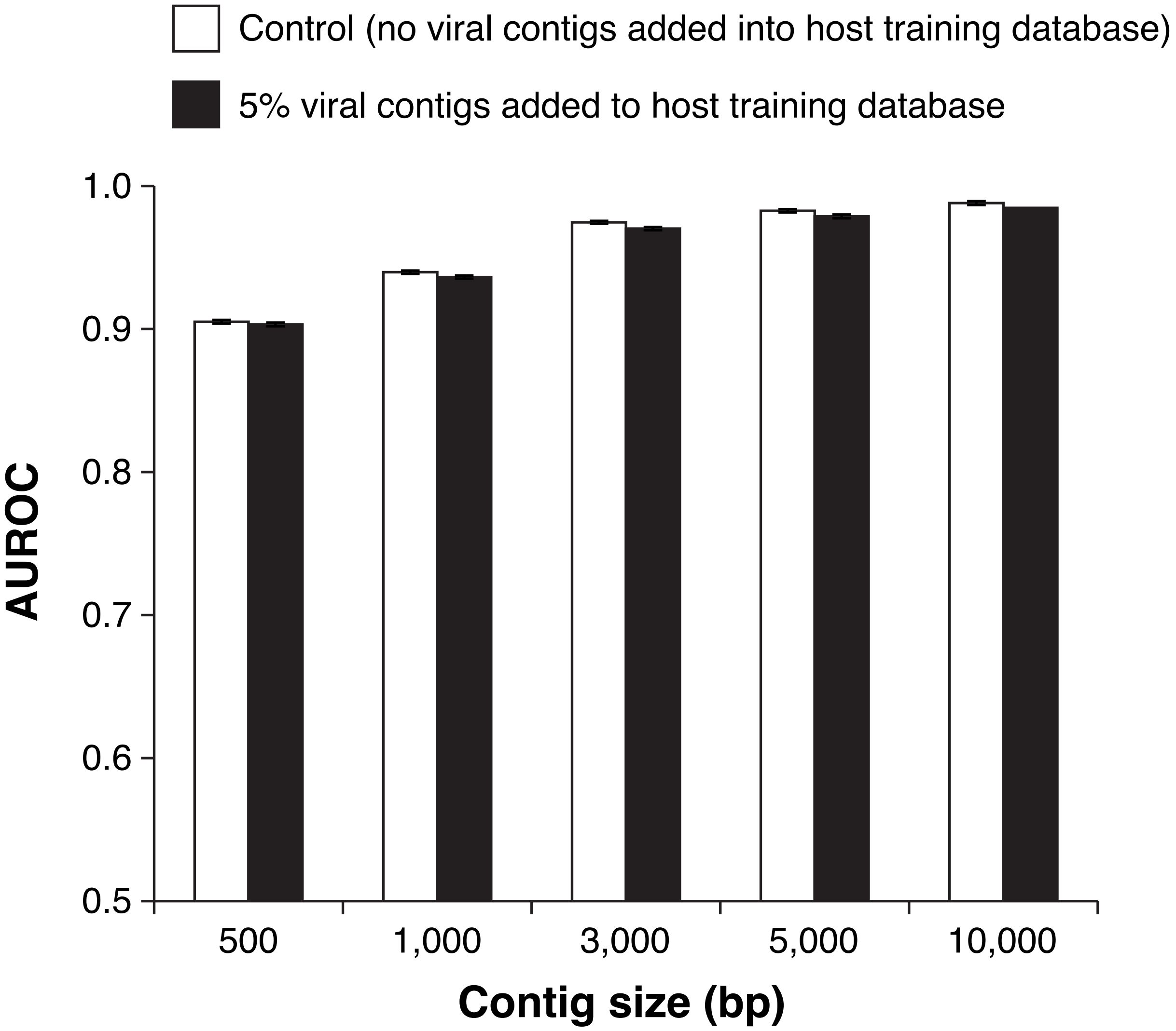

Control (no viral contigs added into host training database)
5% viral contigs added to host training database
1.0
0.9
0.8
0.7
0.6
0.5
AUROC
500
1,000
3,000
5,000
10,000
Contig size (bp)
